# Rational design of crystalline two-dimensional frameworks with highly complicated topological structures

**DOI:** 10.1038/s41467-019-12596-6

**Published:** 2019-10-10

**Authors:** Rong-Ran Liang, Shun-Qi Xu, Lei Zhang, Ru-Han A, Pohua Chen, Fu-Zhi Cui, Qiao-Yan Qi, Junliang Sun, Xin Zhao

**Affiliations:** 1 Key Laboratory of Synthetic and Self-Assembly Chemistry for Organic Functional Molecules, Center for Excellence in Molecular Synthesis, Shanghai Institute of Organic Chemistry, University of Chinese Academy of Sciences, Chinese Academy of Sciences, 345 Lingling Road, Shanghai, 200032 China; 2 Beijing National Laboratory for Molecular Science (BNLMS), State Key Laboratory of Rare Earth Materials Chemistry and Applications, College of Chemistry and Molecular Engineering, Peking University, Beijing, 100871 China

**Keywords:** Organic molecules in materials science, Polymers, Structural properties

## Abstract

Constructing two-dimensional (2D) polymers with complex tessellation patterns via synthetic chemistry makes a significant contribution not only to the understanding of the emergence of complex hierarchical systems in living organisms, but also to the fabrication of advanced hierarchical materials. However, to achieve such tasks is a great challenge. In this communication we report a facile and general approach to tessellate 2D covalent organic frameworks (COFs) by three or four geometric shapes/sizes, which affords 2D COFs bearing three or four different kinds of pores and increases structural complexity in tessellations of 2D polymers to a much higher level. The complex tessellation patterns of the COFs are elucidated by powder X-ray diffraction studies, theoretical simulations and high-resolution TEM.

## Abstract (web summary)

Constructing two-dimensional (2D) polymers with complex tessellation patterns is important for the fabrication of advanced materials but achieving such complexity is a great challenge. Here the authors report a tessellated 2D covalent organic framework with increased structural complexity as well as different pore structures.

## Introduction

The existence of complex hierarchical structures widely found in biological materials is not only one of the most miraculous phenomena in the nature, but also a key foundation for biomaterials to achieve functions^1,2^. With an aim to mimic natural materials in functions as well as in structures, fabrication of materials with hierarchical structures has drawn considerable attention and great progress has been achieved over the past decades^3–5^. However, so far the researches have mainly focused on zero-dimensional (0D), one-dimensional (1D), and three-dimensional (3D) hierarchical materials^6–10^. In terms of two-dimensional (2D) hierarchical structures, they are closely related to an intriguing subject in mathematics, namely tessellation, which refers to completely tiling a plane by one or more geometric shapes without overlaps and gaps^11^. Tessellation patterns are not only widely observed in the nature, but also frequently used in art, architecture and manufacturing. However, the development of tessellations in 2D materials is just in the bud. As the archetype of 2D materials, graphene exhibits a simple hexagonal tilling^12,13^. Similarly, simple tessellation patterns are also observed for the other inorganic 2D materials such as MoS_2_, h-BN and phosphorene^14,15^, due to the limitation of their structural motifs. On the other hand, 2D polymers, which are composed entirely of organic building blocks, provide tremendous structural variabilities. In this context, 2D covalent organic frameworks (COFs), an emerging class of crystalline porous materials precisely integrated with periodical organic units through covalent bonds in a plane and interlayer stacking to form layered 3D crystals via non-covalent interactions^16,17^, have been widely recognized as general 2D polymers, while a stricter definition of 2D polymers refers to those isolated as monolayers^18,19^.

Thanks to the advantages of their conjugated skeletons and porosities, 2D COFs have been extensively exploited to be functional materials with versatile applications ranging from gas separation and storage^20–25^, catalysis^26–30^, drug delivery^31,32^, sensing^33–35^, proton conduction^36,37^, to optoelectronic devices^38–44^. However, despite the progress achieved, the topological structures of 2D COFs are still limited to simple tessellation patterns, which are mainly tiled by only one geometric shape (usually hexagon or tetragon), leading to COFs with homogeneous porosities^45–47^. In 2014, we reported a Kagome-type 2D COF in which hexagonal mesopores and triangular micropores alternately and periodically distribute^48^. Following this dual-pore COF, triple-pore COFs were successful achieved in 2016, which increased the complexity in the tessellations of 2D COFs to a higher level^49^. Such heteropore COFs possess hierarchical structures and exhibit heterogeneous porosities, which benefit for exploiting new properties, functions and applications^50–57^. For example, making use of the advantage that the different kinds of pores can be independently modified and functionalized, distinct functions can be introduced into different types of pores and thus advanced materials with integrated functions can be fabricated. Moreover, different guest molecules, even those incompatible with each other, might be encapsulated in the different types of pores of one COF due to their different pore environments. On the other hand, hierarchically porous materials have already been found to exhibit some advantages over homogeneous ones, such as minimizing diffusion barriers, improving mass transport, and increasing distribution of active sites^3,4^. Indeed, the inimitable advantages of COFs with hierarchical porosity were demonstrated in recent research led by Ma, which clearly showed that a Kagome-shaped dual-pore COF outperformed the COF with uniform porosity in enzyme catalytic performance by loading the enzyme in the large hexagonal pores while making the small triangular pores free to transport reactants and products^58^. Despite the great potential applications, COFs with complex tessellation patterns have remained very difficult to achieve.

We herein report a facile and general approach to fabricate 2D COFs which bear three or four different kinds of pores in one framework. To the best of our knowledge, the latter is the highest level of hierarchy and complexity held by a 2D COF reported so far, demonstrating a successful step further to challenge the structural complexity in the tessellations of 2D polymers.

## Results

### Design and synthesis of the COFs

**Fig. 1:**
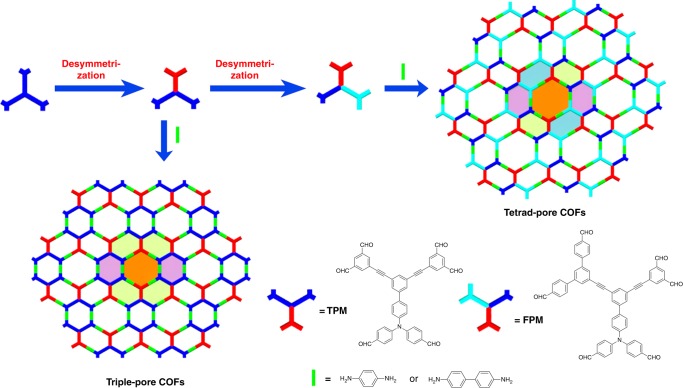
Design strategy. Cartoon representation of the fabrication of triple-pore and tetrad-pore COFs

The approach has been developed by taking advantage of multiple-linking-site^59^ and desymmetrization^60^ strategies simultaneously. As illustrated in Fig. 1, desymmetrization of a C_3_-symmetric double-linking-site building block leads to a monomer with C_2_-symmetry. The condensation of it with linear monomers is expected to afford COFs bearing three different kinds of pores (Fig. 1, lower left). Further desymmetrizing this monomer produces an inequilateral building block, which could be used to tessellate 2D frameworks with four different kinds of pores through assembling it with linear linkers (Fig. 1, right).

Directed by this strategy, monomers TPM and FPM were designed and synthesized (Supplementary Fig. 1). 1,4-diaminobenzene (DAB) and benzidine (BZ) were chosen as the linear monomers. On the basis of the above design, triple-pore 2D COFs (Tri-COF-DAB and Tri-COF-BZ) and tetrad-pore 2D COFs (Tetra-COF-DAB and Tetra-COF-BZ) will be produced by the condensations of TPM or FPM with the diamines, respectively (see Supplementary Fig. 2 for their structures). Tri-COF-DAB was synthesized as yellow crystallites by heating TPM and DAB in a mixture of 1,4-dioxane, mesitylene and acetic acid (aq., 6 M) (5/5/1, v/v/v) in a sealed glass ampoule at 120 °C for 3 days. Under the similar solvothermal condition, Tetra-COF-DAB was prepared from the condensation of FPM and DAB.

### Spectroscopy and thermal stability analysis of the COFs

Fourier transform infrared spectroscopy (FTIR) confirmed formation of imine bonds in the polymers, as evidenced by the appearance of vibration bands of C=N units in their spectra (Supplementary Figs. 3, 4). High degrees of polymerizations of the condensation reactions were indicated by the dramatic attenuation of the vibration bands of NH_2_ groups of DAB. Compared with TPM and FPM, the intensities of C=O stretching vibration peaks of the COFs attenuated dramatically, further confirming the almost complete conversion of the monomers. ^13^C cross-polarization magic angle spinning solid-state NMR spectroscopy was performed for the COFs to characterize the connections of their frameworks. The formation of imines was indicated by the resonance signals of C=N bonds, which were observed at 157.3 and 155.8 ppm in the spectra of Tri-COF-DAB and Tetra-COF-DAB, respectively (Supplementary Figs. 5, 6). Thermogravimetric analysis (TGA) profiles show less than 4.3% weight loss when the temperature increased to 400 °C, indicating good thermal stabilities of these polymers (Supplementary Fig. 7).

### PXRD analysis and structural modeling of the COFs

**Fig. 2:**
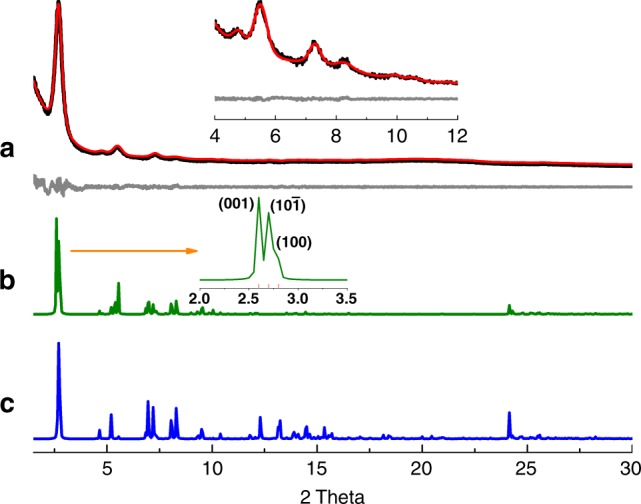
PXRD profiles of Tri-COF-DAB**. a** Experimental (black) and Pawley refined (red) PXRD patterns and difference plot (gray) between them, **b** simulated PXRD patterns with AA stacking, and **c** AB stacking

To elucidate the structure of Tri-COF-DAB, the model of the triple-pore COF with monolayer was firstly predicted on the basis of reticular chemistry by Accelarys Materials Studio 7.0 software. Then two possible stacking models of the COF layers were established and optimized (Supplementary Fig. 8): (i) eclipsed stacking (AA, space group *P*2), (ii) staggered stacking (AB, space group *P*1), based on which the simulated powder X-ray diffraction (PXRD) patterns were obtained (Fig. 2b, c). As for the experimental PXRD profile of Tri-COF-DAB, an intense peak is observed at 2θ = 2.69°, accompanied by a series of relatively weak peaks appearing at 4.73°, 5.47°, 7.23°, and 8.29° (Fig. 2a). It should be pointed out that, in its simulated PXRD pattern with AA stacking, three peaks corresponding to (0 0 1), (1 0 ${\overline{1}}$) and (1 0 0) reflections are very close to each other (Fig. 2b inset and Supplementary Fig. 9a). Due to the broadening and overlapping of these peaks, the three peaks could not be distinguished each other in the experimental PXRD pattern and thus just appear as a single peak centered at 2.69°. A close comparison indicates that the experimental PXRD pattern is in good agreement with that of the simulated triple-pore COF with AA stacking model. Furthermore, the lower total energy of AA stacking (242.72 kcal/mol) than that of AB stacking (515.14 kcal/mol) also indicates that Tri-COF-DAB adopts AA stacking. Pawley refinement was performed to yield unit cell parameters of *a* = 36.79 Å, *b* *=* 3.68 Å, *c* = 39.23 Å, *α* = *γ* = 90°, *β* = 120°, with *R*_wp_ = 3.16% and *R*_p_ = 2.36%. The coincidence of the refined PXRD pattern and experimental data could be evaluated by the difference plot. As revealed by Fig. 2a, the refined PXRD pattern matches with the experimentally obtained PXRD pattern quite well. Based on the PXRD results, the triple-pore structure of Tri-COF-DAB is confirmed.

**Fig. 3:**
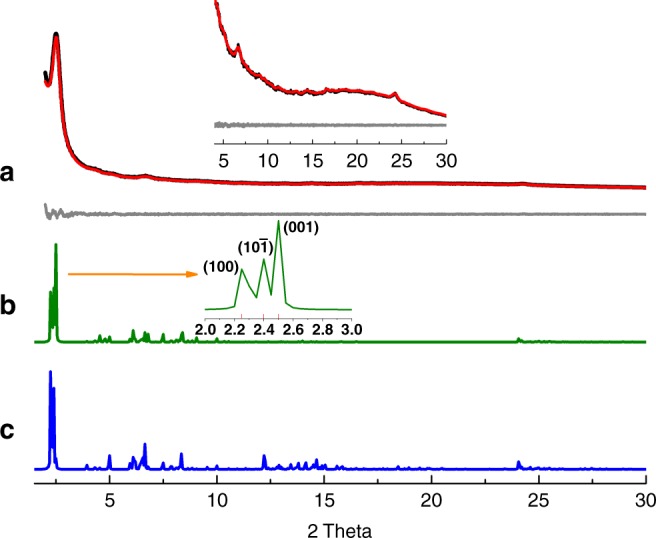
PXRD profiles of Tetra-COF-DAB**. a** Experimental (black) and Pawley refined (red) PXRD patterns and difference plot (gray) between them, **b** simulated PXRD patterns with AA stacking, and **c** AB stacking

The crystal structure of Tetra-COF-DAB was elucidated by the same process as above. It exhibits a strong peak at 2.51°, along with several weak peaks at 5.19°, 6.66°, 9.00°, and 24.25° (Fig. 3a). Its experimental PXRD pattern matches well with the simulated PXRD pattern of the predicted tetrad-pore COF with AA stacking (Fig. 3b), confirming that Tetra-COF-DAB holds a structure bearing four different kinds of pores. The lower total energy of AA stacking (265.64 kcal/mol) than that of AB stacking (623.67 kcal/mol) further supports that Tetra-COF-DAB adopts AA stacking. Pawley refinement gave lattice parameters of *a* = 44.94 Å, *b* = 3.69 Å, *c* = 40.89 Å, *α* = *γ* = 90°, *β* = 120°, with *R*_wp_ = 1.59% and *R*_p_ = 1.03%. The difference plot indicates that PXRD pattern obtained through Pawley refinement well reproduces the experimental pattern (Fig. 3a).

**Fig. 4:**
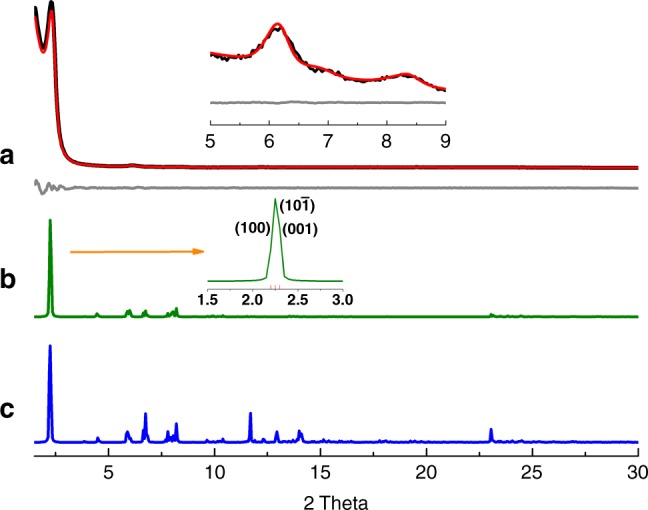
PXRD profiles of Tri-COF-BZ**. a** Experimental (black) and Pawley refined (red) PXRD patterns and difference plot (gray) between them, **b** simulated PXRD patterns with AA stacking, and **c** AB stacking

**Fig. 5:**
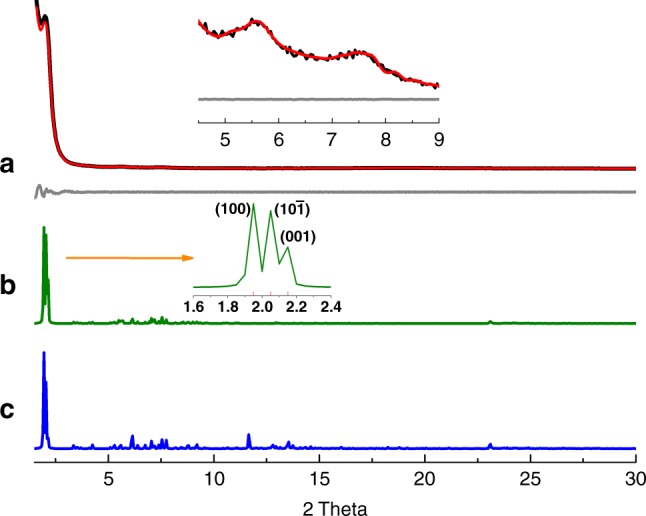
PXRD profiles of Tetra-COF-BZ**. a** Experimental (black) and Pawley refined (red) PXRD patterns and difference plot (gray) between them, **b** simulated PXRD patterns with AA stacking, and **c** AB stacking

To illustrate the generality of this approach, another two triple-pore and tetrad-pore COFs with larger pores, namely Tri-COF-BZ and Tetra-COF-BZ, were also fabricated from the condensations of benzidine with TPM and FPM, respectively (Supplementary Fig. 2). Obtention of the expected COFs bearing three or four different kinds of pores has also been confirmed by comparing the experimental PXRD patterns with the simulated patterns of the predicted models of triple-pore and tetrad-pore COFs (Figs. 4–5). The lattice parameters of Tri-COF-BZ were yielded by Pawley refinement to be *a* = 45.95 Å, *b* = 3.85 Å, *c* = 44.59 Å, *α* = *γ* = 90°, *β* = 120°, with *R*_wp_ = 1.57%, and *R*_p_ = 1.08%. For Tetra-COF-BZ, the unit cell parameters of *a* = 52.50 Å, *b* = 3.85 Å, *c* = 47.86 Å, *α* = *γ* = 90°, *β* = 120°, with *R*_wp_ = 1.50% and *R*_p_ = 1.08% were achieved. The compositions of the two COFs were intensively characterized with FTIR and solid-state CP/MAS ^13^C NMR spectroscopies, the results of which well support the formation of imine-linked polymers with high degrees of polymerizations (Supplementary Figs. 10–13).

### SEM and TEM analysis of the COFs

**Fig. 6:**
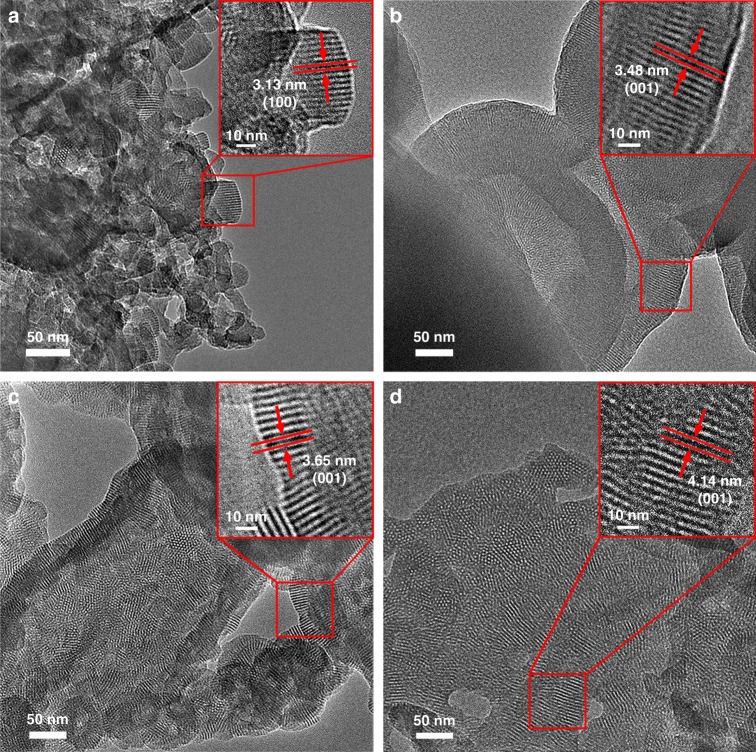
TEM analysis. TEM images of **a** Tri-COF-DAB, **b** Tetra-COF-DAB, **c** Tri-COF-BZ, and **d** Tetra-COF-BZ. The insets in the upper right corners are magnified images from their corresponding red squares

Scanning electron microscopy (SEM) and transmission electron microscopy (TEM) were further conducted to investigate the morphologies and interior structures of the as-obtained COFs. SEM images showed spherical morphologies for all the four COFs (Supplementary Fig. 14). TEM images also indicated the formation of spherical aggregates (Supplementary Fig. 15), which match well with the results of the SEM study. A close analysis of the TEM images revealed that each sphere is polycrystalline, which consists of many 2D flakes. All of these flakes are resolved to be single crystals in good crystallinity by clear lattice fringes (Fig. 6), further indicating high crystallinities of these COFs.

To verify the reliability of the structural identifications of the COFs, which are based on the PXRD results above, detailed TEM study was carried out. Firstly, the fine structures of the lattice fringes in the TEM images were further examined. As shown in Fig. 6, the distances between the lattice fringes were measured to be 3.13, 3.48, 3.65, and 4.14 nm for Tri-COF-DAB, Tetra-COF-DAB, Tri-COF-BZ, and Tetra-COF-BZ, respectively, which are in good agreement with the *d*-spacing of (0 0 1) or (1 0 0) reflection of the predicted triple-pore and tetrad-pore COF models with AA stacking (3.19, 3.54, 3.86, and 4.14 nm for Tri-COF-DAB, Tetra-COF-DAB, Tri-COF-BZ, and Tetra-COF-BZ, respectively), indicating the validity of the predicted COF structures.

**Fig. 7:**
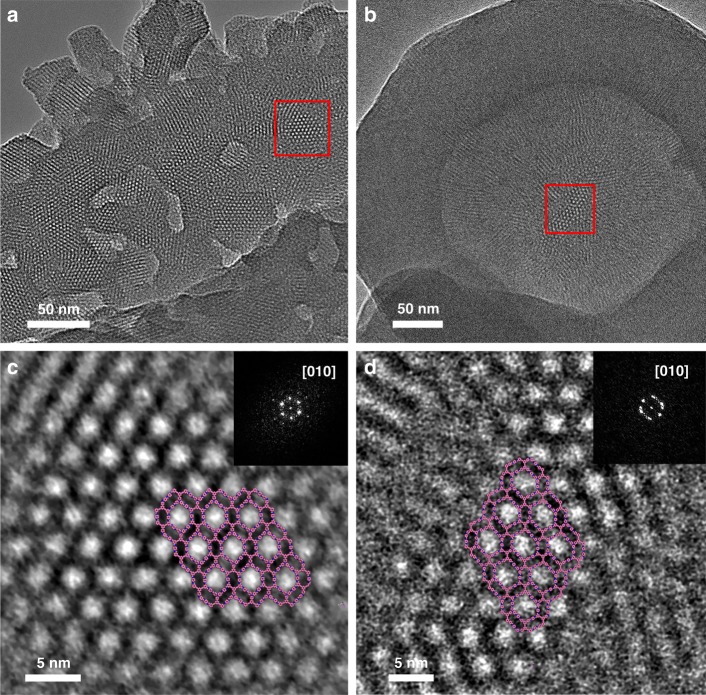
TEM analysis. TEM images of **a** Tri-COF-DAB and **b** Tetra-COF-DAB, and HR-TEM images of **c** Tri-COF-DAB and **d** Tetra-COF-DAB focusing on the regions marked by the red squares in **a** and **b** at a higher resolution. The images inserted in the upper right corners are their corresponding FFT patterns, and the structure projections in HR-TEM images are along the *b*-axis and the structural models are inserted in the middle

**Fig. 8:**
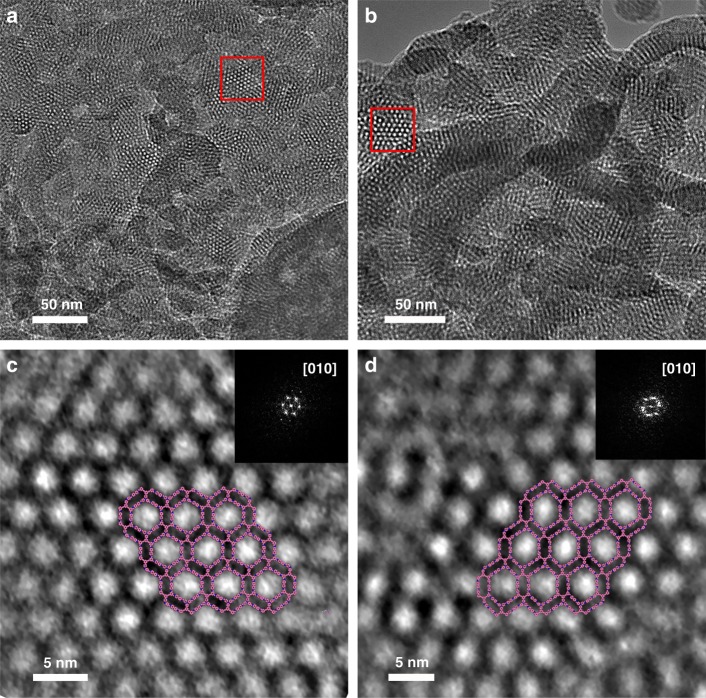
TEM analysis. TEM images of **a** Tri-COF-BZ and **b** Tetra-COF-BZ, and HR-TEM images of **c** Tri-COF-BZ and **d** Tetra-COF-BZ focusing on the regions marked by the red squares in **a** and **b** at a higher resolution. The images inserted in the upper right corners are their corresponding FFT patterns, the structure projections in HR-TEM images are along the *b*-axis and the structural models are inserted in the middle

Furthermore, as shown in Figs. 7a, b and 8a, b, network structures could be observed in the TEM images of these four COFs. To obtain clearer visualizations of the networks, high-resolution (HR) TEM images of the regions marked by the red squares in Figs. 7a, b and Fig. 8a, b were further recorded at a higher resolution to produce Figs. 7c, d and 8c, d, respectively. As shown in these figures, clear reticular structures with approximately hexagonal pores could be observed. The distances between the centers of the two adjacent pores are 3.5 ± 0.1 nm, 4.1 ± 0.1 nm, 4.2 ± 0.1 nm, and 4.8 ± 0.2 nm for Tri-COF-DAB, Tetra-COF-DAB, Tri-COF-BZ, and Tetra-COF-BZ, respectively, which are in good agreement with the unit cell of *a* or *c* derived from the Pawley refinements of the PXRD data, indicating that the observed crystal structures of the four COFs match well with the theoretical structure models along the *b*-axis. The images inserted in the upper right corners are the corresponding diffraction patterns of the four COFs obtained through fast Fourier transformation (FFT), further indicating single-crystal characteristics of these 2D COF flakes. The TEM investigation again corroborates that the as-prepared COFs hold the predicted triple-pore and tetrad-pore framework structures.

### Surface area and porosity measurements of the COFs

**Fig. 9:**
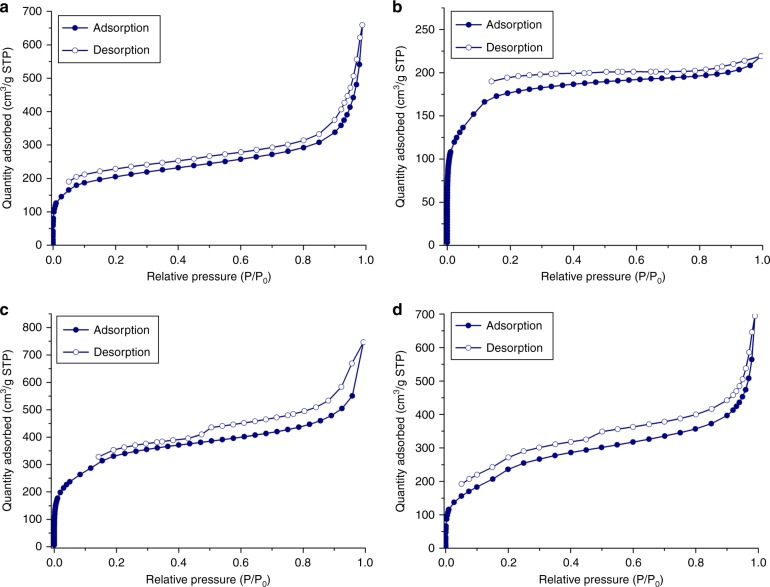
Porosity measurements. N_2_ adsorption−desorption isotherms (77 K) of **a** Tri-COF-DAB, **b** Tetra-COF-DAB, **c** Tri-COF-BZ, and **d** Tetra-COF-BZ

Porosities of the COFs were assessed by nitrogen adsorption-desorption measurements (Fig. 9). Their BET surface areas, derived from their nitrogen adsorption isotherms at 77 K, are 673.8, 1180.8, 568.8, and 864.3 m^2^ g^−1^ for Tri-COF-DAB, Tri-COF-BZ, Tetra-COF-DAB, and Tetra-COF-BZ, respectively (Supplementary Fig. 16), and their corresponding total pore volumes (at P/P_o_ = 0.99) were estimated to be 1.02, 1.15, 0.34, and 1.07 cm^3^ g^−1^. Derived from their theoretical N_2_ adsorption isotherms (Supplementary Fig. 17), the theoretical BET surface areas were calculated to be 1659.8, 2388.4, 1900.7, and 2312.8 m^2^ g^−1^ for Tri-COF-DAB, Tri-COF-BZ, Tetra-COF-DAB, and Tetra-COF-BZ, respectively (Supplementary Fig. 18). The low experimental values are likely attributed to framework flexibility of the COFs resulting from their large unit cell dimensions^61^. It should be noted that no informative information could be obtained from pore size distribution (PSD) analysis because no suitable model could be applied to these highly complexed COFs. Taking Tetra-COF-BZ as an example, PSD analysis was carried out by trying all the kernels/pore models available from the instrument. However, not an appropriate model was found to be suitable for the COF, as the PSD profiles obtained did not have a definite meaning to the COFs (Supplementary Fig. 19), which may be due to the defects of the COFs or other effects. This result suggests the limitation of PSD on such complex framework materials. However, although PSD data are not available, the results of PXRD and HR-TEM provide compelling evidence for the formation of the pre-designed triple-pore and tetrad-pore COFs.

## Discussion

In summary, a facile and general approach has been developed to construct 2D COFs with highly complicated topological structures. Implementation of this approach led to the successful construction of 2D COFs for which three or four different kinds of pores were integrated into one framework, increasing the structural complexity in the tessellations of 2D polymers to a much higher level. This work also demonstrates that the mathematically intricate tessellation patterns could be successfully challenged by synthetic chemistry. Since tessellating 2D COFs with different geometric shapes provides access to independent structural modification and functionalization of different parts of frameworks with high precision, it opens a promising way to fabricate advanced materials in which different functions can be highly integrated. With the development of more elaborate tessellation strategies, emergence of 2D polymers with much more structural complexity and multiple-functionalities can be expected in the near future.

## Methods

### Synthesis of Tri-COF-DAB

TPM (30.0 mg, 0.043 mmol) and 1,4-diaminobenzene (DAB, 14.1 mg, 0.130 mmol) were added into a mixture of 1,4-dioxane (0.75 mL), mesitylene (0.75 mL), and acetic acid (aq. 6 M, 0.15 mL) in a glass ampoule. The ampoule was sealed under vacuum after three freeze-pump-thaw cycles. Then the mixture was heated at 120 °C without disturbance for 72 h to afford a yellow solid at the bottom of the tube. After being cooled to room temperature, the solvent was decanted and the solid was washed with anhydrous 1,4-dioxane and DCM for three times, respectively, and then dried under dynamic vacuum at 120 °C for 4 h to afford a yellow powder (31.7 mg, 81%), which was insoluble in common organic solvents such as acetone, ethanol, and N, N-dimethylformamide. Anal. Calcd. For C_64_H_39_N_7_: C, 84.84; H, 4.34; N, 10.82. Found: C, 79.23; H, 4.75; N, 9.57.

### Synthesis of Tri-COF-BZ

TPM (25.0 mg, 0.036 mmol) and benzidine (BZ, 20.0 mg, 0.108 mmol) were added into a mixture of 1, 4-dioxane (1 mL), mesitylene (1 mL) and acetic acid (aq. 6 M, 0.2 mL) in a glass ampoule. The ampoule was sealed under vacuum after three freeze-pump-thaw cycles. Then the mixture was heated at 120 °C without disturbance for 72 h to afford a yellow solid at the bottom of the tube. After being cooled to room temperature, the solvent was decanted and the solid was washed with anhydrous 1,4-dioxane and DCM for three times, respectively, and then dried under dynamic vacuum at 120 °C for 4 h to afford a yellow powder (30.0 mg, 73%), which was insoluble in common organic solvents such as acetone, ethanol, and N, N-dimethylformamide. Anal. Calcd. For C_82_H_51_N_7_: C, 86.82; H, 4.53; N, 8.64. Found: C, 81.26; H, 4.98; N, 7.50.

### Synthesis of Tetra-COF-DAB

FPM (26.0 mg, 0.031 mmol) and DAB (10.0 mg, 0.092 mmol) were added into a mixture of 1,4-dioxane (0.25 mL), mesitylene (0.75 mL) and acetic acid (aq. 6 M, 0.1 mL) in a glass ampoule. The ampoule was sealed under vacuum after three freeze-pump-thaw cycles. Then the mixture was heated at 120 °C without disturbance for 72 h to afford a yellow solid at the bottom of the tube. After being cooled to room temperature, the solvent was decanted and the solid was washed with anhydrous 1,4-dioxane and DCM for three times, respectively, and then dried under dynamic vacuum at 120 °C for 4 h to afford a yellow powder (19.7 mg, 60%), which was insoluble in common organic solvents such as acetone, ethanol, and N, N-dimethylformamide. Anal. Calcd. For C_76_H_47_N_7_: C, 86.26; H, 4.48; N, 9.27. Found: C, 80.05; H, 5.13; N, 7.02.

### Synthesis of Tetra-COF-BZ

FPM (27.4 mg, 0.032 mmol) and BZ (18.0 mg, 0.098 mmol) were added into a mixture of 1,4-dioxane (1 mL), mesitylene (1 mL) and acetic acid (aq. 9 M, 0.2 mL) in a glass ampoule. The ampoule was sealed under vacuum after three freeze-pump-thaw cycles. Then the mixture was heated at 120 °C without disturbance for 72 h to afford a yellow solid at the bottom of the tube. After being cooled to room temperature, the solvent was decanted and the solid was washed with anhydrous 1,4-dioxane and DCM for three times, respectively, and then dried under dynamic vacuum at 120 °C for 4 h to afford a yellow powder (37.0 mg, 90%), which was insoluble in common organic solvents such as acetone, ethanol, and N, N-dimethylformamide. Anal. Calcd. For C_94_H_59_N_7_: C, 87.76; H, 4.62; N, 7.62. Found: C, 84.79; H, 5.14; N, 7.25.

## Supplementary information

Supplementary Information

## Data Availability

All data supporting the findings of this study are available within the article, as well as the Supplementary Information file, or available from the corresponding authors on reasonable request.
