# Supplementary material for: Rational design of crystalline two-dimensional frameworks with highly complicated topological structures
Source: Nat Commun. 2019 Oct 10;10:4609. doi: 10.1038/s41467-019-12596-6 (PMC6787252; doi:10.1038/s41467-019-12596-6)
Supplement: Supplementary file 1 — Supplementary Information [file 41467_2019_12596_MOESM1_ESM.pdf]

Supplementary Information

Rational design of crystalline two-dimensional frameworks with highly complicated topological structures

Liang *et al.*

Table of contents

Supplementary Methods...S3-S10

Supplementary Figure 1. Synthetic routes to TPM and FPM..................................S5

Supplementary Figure 2. Synthetic procedures of the COFs.................................S11

Supplementary Figures 3-4. FT-IR spectra of the monomers, Tri-COF-DAB and Tetra-COF-DAB...S11-S12

Supplementary Figures 5-6. Solid-state ^{13}C CP/MAS NMR spectra of Tri-COF-DAB and Tetra-COF-DAB...S12-S13

Supplementary Figure 7. TGA profiles of the COFs...S13

Supplementary Figure 8. Illustrations of AA and AB stacking models.................S14

Supplementary Figure 9. The close-up views of the simulated PXRD patterns of the COFs with AA stacking models..S14

Supplementary Figures 10-11. FT-IR spectra of the monomers, Tri-COF-BZ and Tetra-COF-BZ..S15

Supplementary Figures 12-13. Solid-state ^{13}C CP/MAS NMR spectra of Tri-COF-BZ and Tetra-COF-BZ..S16

Supplementary Figure 14. SEM images of the COFs..S17

Supplementary Figure 15. TEM images of the COFs..S17

Supplementary Figure 16. BET surface area plots of the COFs............................S18

Supplementary Figure 17. Theoretical N_2 adsorption−desorption isotherms of the COFs...S18

Supplementary Figure 18. Theoretical BET surface area plots of the COFs..........S19

Supplementary Figure 19. PSD profiles of Tetra-COF-BZ....................................S20

Supplementary Tables 1-4. Fractional atomic coordinates of the COFs..........S20-S30

Supplementary Figures 20-39. ^{1}H NMR and ^{13}C NMR spectra of the new compounds...S31-S40

Supplementary References...S41

Supplementary Methods

Fourier transform infrared spectroscopy (FT-IR)

Fourier transform infrared spectroscopy (FT-IR) was carried out with a Nicolet 380 FT-IR spectrometer. The samples for IR study were prepared as KBr pellets.

Solid-state nuclear magnetic resonance (NMR) spectroscopy

The ^{13}C CP/MAS NMR spectra of the COFs were recorded on an Agilent DD2 600 Solid NMR System with 4 mm zirconia rotors. The spinning rate is 9 kHz and the contact time is 3 ms.

Thermal gravimetric analysis (TGA)

Thermal gravimetric analysis was conducted on a Waters TGA Q500 by heating the samples from 25 to 1000 ℃ under nitrogen atmosphere with a heating rate of 10 ℃/min.

Scanning electron microscopy (SEM)

Scanning electron microscopy was carried out using a FEI NOVA NANOSEM 450 scanning electron microscope. The samples were dispersed over the slices of silicon wafers adhered to flat copper platform sample holders and then coated with gold using a sputter coater (ambient temperature, 85 torr pressure in an nitrogen atmosphere, puttered for 30 s from a solid gold target at a current at 30 mA) before being submitted to SEM characterization.

Transmission electron microscopy (TEM)

Transmission electron microscopy was performed on a JEOL JEM-2100F instrument with an accelerating voltage of 200 kV. The samples were dispersed over the carbon coated copper grids.

Powder X-ray diffraction

Powder X-ray diffraction measurement was carried out with an PANalytical X’Pert

Powder system using monochromated Cu/Kα(λ= 0.1542 nm). The samples were spread on the square recess of XRD sample holder as thin layers.

Nitrogen adsorption-desorption isotherm measurement

The measurements were carried out using Micromeritics ASAP 2020 or Quantanchrome autosorb iq systems. Before gas adsorption measurements, the as-synthesized COFs were activated by being immersed in anhydrous 1,4-dioxane for 6 h for 3 times. The solvent was decanted and the samples were dried under dynamic vacuum at 120 °C for 4 h. The resulting samples were then activated by degassing at 200 ^{o}C for 4 h and used for gas adsorption measurements from 0 to 1 atm at 77 K. The Brunauer-Emmett-Teller (BET) method was utilized to calculate their specific surface areas.

Structural simulation and powder X-ray diffraction analysis

The models of the triple-pore and tetrad-pore COFs with eclipsed stacking (AA, space group *P*2) and staggered stacking (AB, space group *P*1) were established using Accelarys Materials Studio 7.0 software. The structural models were optimized by the Forcite module, which gave the total energies at the same time. The stimulated PXRD patterns were determined by the Reflex module. Pawley refinements of the experimental PXRD profiles were conducted by TOPAS software.

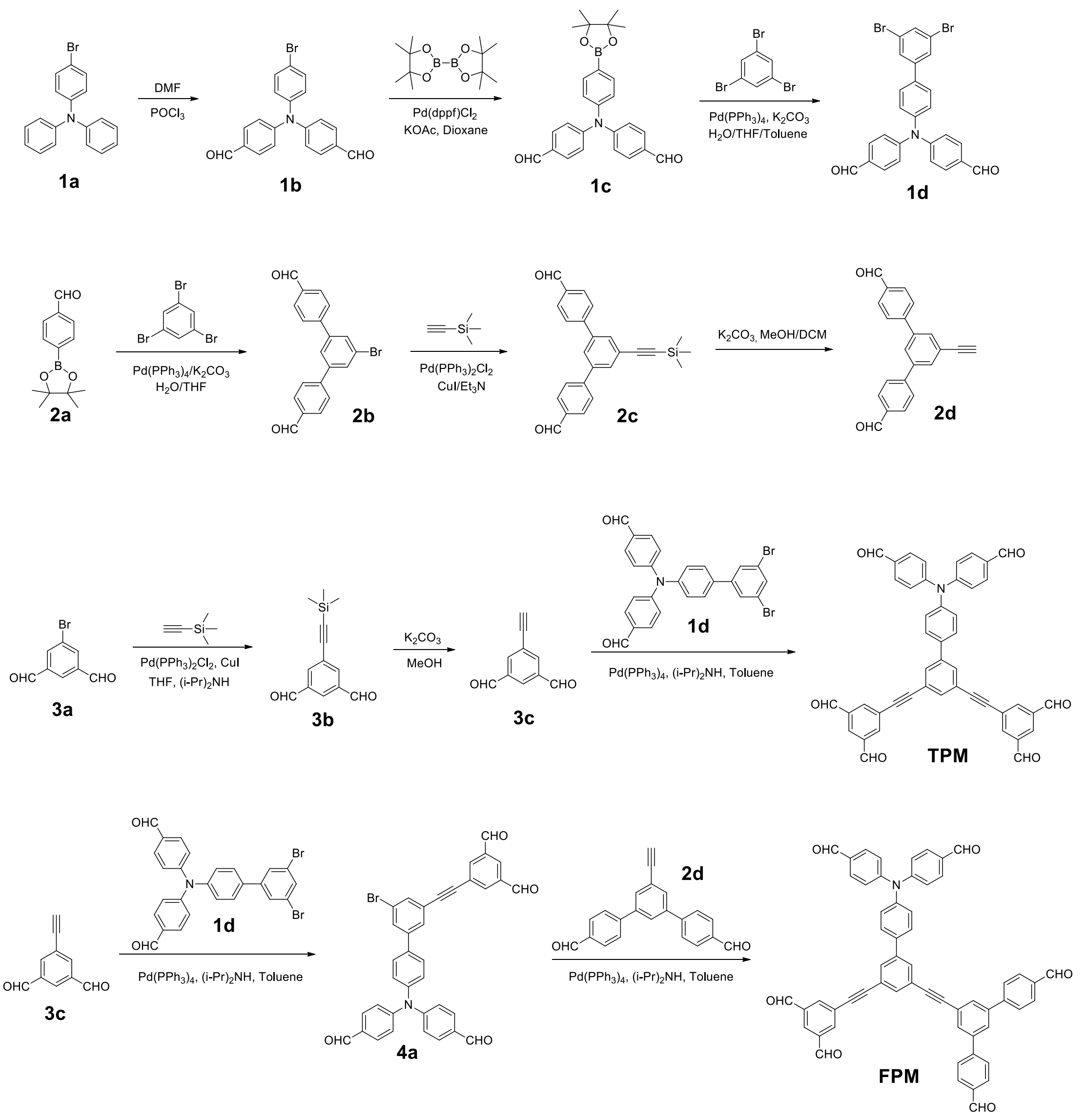

Supplementary Figure 1. Synthetic routes to TPM and FPM.

Synthesis of 1c

Compound 1b (4.00 g, 10.52 mmol, synthesized according to the reported procedure[1]), bis(pinacolato)diboron (4.12 g, 16.22 mmol), Potassium acetate (3.40 g, 34.64 mmol) and Pd(dppf)Cl_2 (0.84 g, 1.15 mmol) were mixed in dioxane (160 mL). The flask was cooled to -78 ^{o}C and subjected to three evacuation/nitrogen fill cycles. The reaction mixture was gradually warmed to room temperature, and stirred at 85 ^{o}C for 24 h under N_2 atmosphere. After cooling to room temperature, the solvent was evaporated to dryness under reduced pressure. And then the crude product was subjected to silica gel chromatography with petroleum ether/CH_2Cl_2 (1/1, v/v) as eluent to give 1c as a

yellow solid (3.73 g, 83%). ^{1}H NMR (500 MHz, $CDCl_3$): δ 9.90 (s, 2H), 7.81 (d, *J* = 8.3 Hz, 2H), 7.78 (d, *J* = 8.6 Hz, 4H), 7.19 (d, *J* = 8.6 Hz, 4H), 7.15 (d, *J* = 8.3 Hz, 2H), 1.36 (s, 12H). ^{13}C NMR (101 MHz, $CDCl_3$): δ 190.75, 152.03, 148.39, 136.77, 131.78, 131.51, 125.77, 123.48, 84.22, 25.07. MS (MALDI): *m/z* 427.0 $[M+H]^+$. HRMS (DART Positive Ion Mode): Calcd. for $C_{26}H_{27}O_4NB$ $[M+H]^+$: 427.2064. Found: 427.2061.

Synthesis of 1d

Compound 1c (3.00 g, 7.02 mmol), tribromobenzene (6.00 g, 19.06 mmol), $Pd(PPh_3)_4$ (0.83 mg, 0.72 mmol) and K_2CO_3 (6.40 g, 46.31 mmol) were mixed in a solution of toluene (100 mL), THF (20 mL) and H_2O (30 mL). The flask was cooled to -78 °C and subjected to three evacuation/nitrogen fill cycles. Then the mixture was refluxed under a nitrogen atmosphere for 48 h at 80 °C. After being cooled to room temperature, the mixture was extracted with $CHCl_2$. The organic layer was washed with water and brine and then dried over sodium sulfate. After removal of the solvents, the residue was purified by silica gel chromatography (eluent: petroleum ether/CH_2Cl_2 = 1/3) to yield 1d as a yellow powder (2.63 g, 70%). ^{1}H NMR (500 MHz, $CDCl_3$): δ 9.92 (s, 2H), 7.81 (d, *J* = 8.7 Hz, 4H), 7.66 (s, 3H), 7.53 (d, *J* = 8.6 Hz, 2H), 7.23 (dd, *J* = 8.5, 6.4 Hz, 6H). ^{13}C NMR (126 MHz, $CDCl_3$): δ 190.68, 151.93, 146.10, 143.76, 135.95, 133.04, 131.91, 131.61, 128.98, 128.94, 127.12, 123.64, 123.42. MS (MALDI): *m/z* 534.1 $[M+H]^+$. HRMS (DART Positive Ion Mode): Calcd. for $C_{26}H_{18}O_2NBr_2$ $[M+H]^+$: 533.9699. Found: 533.9699.

Synthesis of 2b

Compound 2a (3.01 g, 12.97 mmol), tribromobenzene (2.03 g, 6.45 mmol), $Pd(PPh_3)_4$ (1.51 g, 1.31 mmol) and K_2CO_3 (11.87 g, 85.89 mmol) were added in a three-necked, round-bottomed flask. After three evacuation/nitrogen fill cycles, THF (150 mL) and H_2O (30 mL) were injected into the flask under nitrogen atmosphere. Then the mixture was refluxed for 3 days at 75 °C. After being cooled to room temperature, the mixture was extracted with CH_2Cl_2. The organic layer was washed with water and

brine and dried over sodium sulfate. After removal of the solvents, the residue was purified by silica gel chromatography (eluent: petroleum ether/ethyl acetate = 8/1) to yield 2b as a white powder (1.67 g, 71%). ^{1}H NMR (500 MHz, $CDCl_3$): δ 10.09 (s, 2H), 8.00 (d, *J* = 8.1 Hz, 4H), 7.80 (dd, *J* = 14.7, 4.7 Hz, 7H). ^{13}C NMR (126 MHz, $CDCl_3$): δ 191.88, 145.40, 142.73, 136.11, 130.62, 130.42, 128.09, 125.40, 123.97. MS (DART Positive Ion Mode): *m/z* 365.0 $[M+H]^+$. HRMS (DART Positive Ion Mode): Calcd. for $C_{20}H_{14}O_2Br$ $[M+H]^+$: 365.0172. Found: 365.0171.

Synthesis of 2c

A 100 mL two-neck round bottom flask was charged with compound 2b (1.16 g, 3.18 mmol), CuI (31 mg, 0.16 mmol), $Pd(PPh_3)_2Cl_2$ (228 mg, 0.32 mmol) in triethylamine (35 mL) under nitrogen atmosphere. Trimethylsilylacetylene (1.9 mL, 13.44 mmol) was added dropwise to the mixture under high nitrogen flow and the reaction continued at room temperature for 2 days. The solvent was removed under vacuum and the crude product was purified by silica gel chromatography using a mixture of petroleum ether/ethyl acetate = 8/1 as eluent to afford 2c as a yellow solid (0.81 g, 66%). ^{1}H NMR (500 MHz, DMSO-d_6): δ 10.09 (s, 2H), 8.15 (s, 1H), 8.11 (d, *J* = 8.2 Hz, 4H), 8.02 (d, *J* = 8.3 Hz, 4H), 7.90 (d, *J* = 1.6 Hz, 2H), 0.28 (s, 9H). ^{13}C NMR (101 MHz, DMSO-d_6): δ 192.85, 144.34, 140.19, 135.55, 130.08, 130.01, 127.91, 126.65, 123.86, 104.50, 95.32, -0.12. MS (DART Positive Ion Mode): *m/z* 383.1 $[M+H]^+$. HRMS (DART Positive Ion Mode): Calcd. for $C_{25}H_{23}O_2Si$ $[M+H]^+$: 383.1462. Found: 383.1460.

Synthesis of 2d

A mixture of compound 2c (379 mg, 0.99 mmol) and K_2CO_3 (289 mg, 2.09 mmol) was dissolved in a solvent mixture of dichloromethane (6 mL) and methanol (9 mL). After three evacuation/nitrogen fill cycles, the mixture was stirred at room temperature for 24 h. Then, 20 mL water was added into the system, and the mixture was extracted with CH_2Cl_2. The organic layer was washed with brine and dried over sodium sulfate. After removal of the solvents, the crude product was purified by silica

gel chromatography using petroleum ether/ethyl acetate (8/1) as eluent to obtain 2d as a white solid (0.18 g, 58%). ^{1}H NMR (500 MHz, $CDCl_3$): δ 10.09 (s, 2H), 8.00 (d, *J* = 7.9 Hz, 4H), 7.84 (s, 1H), 7.82 – 7.78 (m, 6H), 3.20 (s, 1H). ^{13}C NMR (126 MHz, $CDCl_3$): δ 191.94, 145.88, 141.13, 135.97, 131.04, 130.61, 128.04, 127.01, 124.00, 82.97, 78.64. MS (DART Positive Ion Mode): *m/z* 311.1 $[M+H]^+$. HRMS (DART Positive Ion Mode): Calcd. for $C_{22}H_{15}O_2$ $[M+H]^+$: 311.1067. Found: 311.1066.

Synthesis of 3b

A 250 mL three-necked round-bottomed flask was charged with compound 3a (9.20 g, 43.19 mmol), CuI (250 mg, 1.31 mmol), $Pd(PPh_3)_2Cl_2$ (910 mg, 1.30 mmol) in THF (100 mL) and diisopropylamine (45 mL) under nitrogen atmosphere and stirred for 5 min at room temperature. Trimethylsilylacetylene (11 mL, 77.81 mmol) was added dropwise to the mixture under high nitrogen flow and the reaction continued at room temperature for 3 h. The solvent was removed under vacuum and the crude product was purified by silica gel chromatography using a mixture of petroleum ether/CH_2Cl_2 (4/1) as eluent to afford 3b as a light yellow solid (6.37 g, 64%). ^{1}H NMR (500 MHz, $CDCl_3$): δ 10.08 (s, 2H), 8.30 (t, *J* = 1.5 Hz, 1H), 8.19 (d, *J* = 1.5 Hz, 2H), 0.28 (s, 9H). ^{13}C NMR (101 MHz, $CDCl_3$): δ 167.82, 125.73, 125.24, 119.32, 116.20, 97.08, 94.39, 15.39. MS (DART Positive Ion Mode): *m/z* 231.1 $[M+H]^+$. HRMS (DART Positive Ion Mode): Calcd. for $C_{13}H_{15}O_2Si$ $[M+H]^+$: 231.0836. Found: 231.0835.

Synthesis of 3c

A mixture of compound 3b (2.14 g, 9.29 mmol) and K_2CO_3 (1.28 g, 9.26 mmol) was dissolved in methanol (60 mL). After three evacuation/nitrogen fill cycles, the mixture was stirred at room temperature for 3 h. Then, 50 mL water was added into the system, and the mixture was extracted with CH_2Cl_2. The organic layer was washed with brine and dried over sodium sulfate. After removal of the solvents, the crude product was purified by silica gel chromatography (petroleum ether/CH_2Cl_2 = 1/1) to give 3c as a white solid (1.44 g, 98%). ^{1}H NMR (500 MHz, $CDCl_3$): δ 10.09 (s, 2H), 8.35 (t, *J* = 1.4 Hz, 1H), 8.23 (d, *J* = 1.5 Hz, 2H), 3.27 (s, 1H). ^{13}C NMR (126 MHz, $CDCl_3$): δ

190.30, 138.00, 137.34, 130.34, 124.93, 81.03, 80.76. MS (DART Positive Ion Mode): *m/z* 159.0 $[M+H]^+$. HRMS (DART Positive Ion Mode): Calcd. for $C_{10}H_7O_2$ $[M+H]^+$: 159.0441. Found: 159.0440.

Synthesis of 4a

Compound **3c** (590 mg, 3.73 mmol), compound 1d (2.00 g, 3.74 mmol) and $Pd(PPh_3)_4$ (646 mg, 0.56 mmol) were mixed in toluene (105 mL) and diisopropylamine (35 mL). Then the mixture was subjected to three evacuation/nitrogen fill cycles and heated at 80 °C for 2 days under nitrogen atmosphere. After the solution cooled down, the ammonium salt was filtered. The filtrate was evaporated and the residue was purified by silica gel chromatography (petroleum ether/ethyl acetate = 3/1) to give 4a as a yellow solid (1.27 g, 56%). ^{1}H NMR (500 MHz, $CDCl_3$): δ 10.12 (s, 2H), 9.93 (s, 2H), 8.35 (t, J = 1.5 Hz, 1H), 8.28 (d, J = 1.5 Hz, 2H), 7.82 (d, J = 8.7 Hz, 4H), 7.76 (t, J = 1.7 Hz, 1H), 7.72 (t, J = 1.5 Hz, 1H), 7.71 – 7.70 (m, 1H), 7.59 (d, J = 8.7 Hz, 2H), 7.27 (s, 1H), 7.26 (s, 1H), 7.24 (d, J = 8.6 Hz, 4H). ^{13}C NMR (126 MHz, $CDCl_3$): δ 190.69, 190.34, 151.96, 146.04, 142.49, 137.47, 137.40, 136.34, 133.37, 131.62, 130.32, 129.13, 128.94, 127.18, 125.46, 124.71, 123.42, 123.14, 90.94, 88.20. MS (MALDI): *m/z* 612.0 $[M+H]^+$. HRMS (DART Positive Ion Mode): Calcd. for $C_{36}H_{23}O_4NBr$ $[M+H]^+$: 612.0805. Found: 612.0800.

Synthesis of TPM

Compound 3c (122 mg, 0.77 mmol), compound 1d (104 mg, 0.19 mmol) and $Pd(PPh_3)_4$ (34 mg, 0.029 mmol) were mixed in toluene (30 mL) and diisopropylamine (10 mL). Then the mixture was subjected to three evacuation/nitrogen fill cycles and heated at 80 °C for 2 days under nitrogen atmosphere. After the solution was cooled down to room temperature, the ammonium salt was filtered. The filtrate was evaporated and the residue was purified by silica gel chromatography (CH_2Cl_2/ethyl acetate = 10/1) to give TPM as a yellow solid (83 mg, 62%). ^{1}H NMR (500 MHz, $CDCl_3$): δ 10.13 (s, 4H), 9.93 (s, 2H), 8.36 (d, J = 1.4 Hz, 2H), 8.30 (d, J = 1.4 Hz, 4H), 7.83 (d, J = 8.7 Hz, 6H), 7.76 (s, 1H), 7.66 (d, J = 8.5 Hz, 2H), 7.29 (d, J = 8.6

Hz, 2H), 7.26 (d, *J* = 7.9 Hz, 4H). ^{13}C NMR (126 MHz, $CDCl_3$): δ 190.70, 190.37, 151.98, 145.96, 141.23, 137.46, 137.37, 136.68, 133.79, 131.88, 131.61, 130.92, 130.34, 128.92, 127.22, 125.56, 123.63, 123.40, 91.38, 87.93. MS (MALDI): *m/z* 689.1 $[M]^+$. HRMS (DART Positive Ion Mode): Calcd. for $C_{46}H_{27}O_6N$ $[M]^+$: 689.1833. Found: 689.1831.

Synthesis of FPM

Compound 4a (480 mg, 0.78 mmol), compound 2d (240 mg, 0.77 mmol) and $Pd(PPh_3)_4$ (113 mg, 0.098 mmol) were mixed in toluene (36 mL) and diisopropylamine (12 mL). Then the mixture was subjected to three evacuation/nitrogen fill cycles and heated at 80 °C for 2 days under nitrogen atmosphere. After the solution cooled down, the ammonium salt was filtered, the filtrate was evaporated and the residue was purified by silica gel chromatography (CH_2Cl_2/ethyl acetate = 30/1) to give FPM as a yellow solid (209 mg, 32%). ^{1}H NMR (500 MHz, $CDCl_3$): δ 10.13 (s, 2H), 10.10 (s, 2H), 9.93 (s, 2H), 8.35 (s, 1H), 8.30 (s, 2H), 8.02 (d, *J* = 8.0 Hz, 4H), 7.89 – 7.85 (m, 5H), 7.85 – 7.81 (m, 7H), 7.79 (d, *J* = 9.4 Hz, 2H), 7.66 (d, *J* = 8.3 Hz, 2H), 7.29 (d, *J* = 8.3 Hz, 2H), 7.26 (d, *J* = 8.5 Hz, 4H). ^{13}C NMR (126 MHz, $CDCl_3$): δ 191.90, 190.68, 190.37, 151.97, 145.87, 141.25, 141.11, 137.44, 137.31, 136.82, 136.00, 133.76, 131.85, 131.59, 130.85, 130.62, 130.56, 130.45, 130.33, 128.90, 128.04, 127.21, 126.86, 125.59, 124.60, 124.27, 123.50, 123.36, 91.55, 89.97, 89.47, 87.79. MS (MALDI): *m/z* 842 .4 $[M+H]^+$. HRMS (DART Positive Ion Mode): Calcd. for $C_{58}H_{36}O_6N$ $[M+H]^+$: 842.2537. Found: 842.2531.

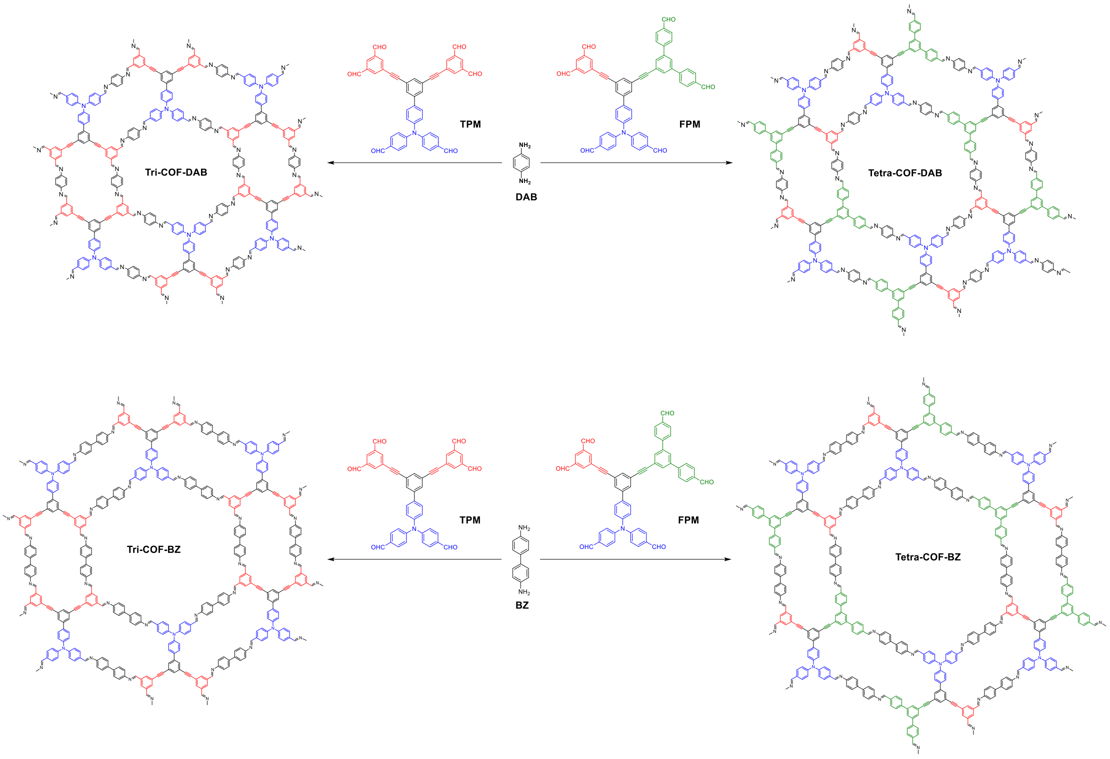

Supplementary Figure 2. Synthetic procedures of the COFs.

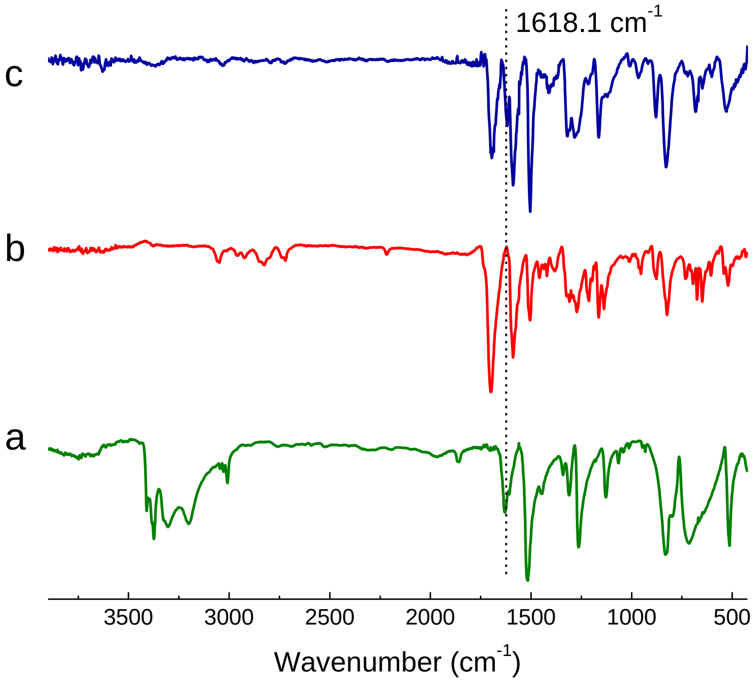

Supplementary Figure 3. FT-IR spectra of **a** DAB, **b** TPM, and **c** Tri-COF-DAB.

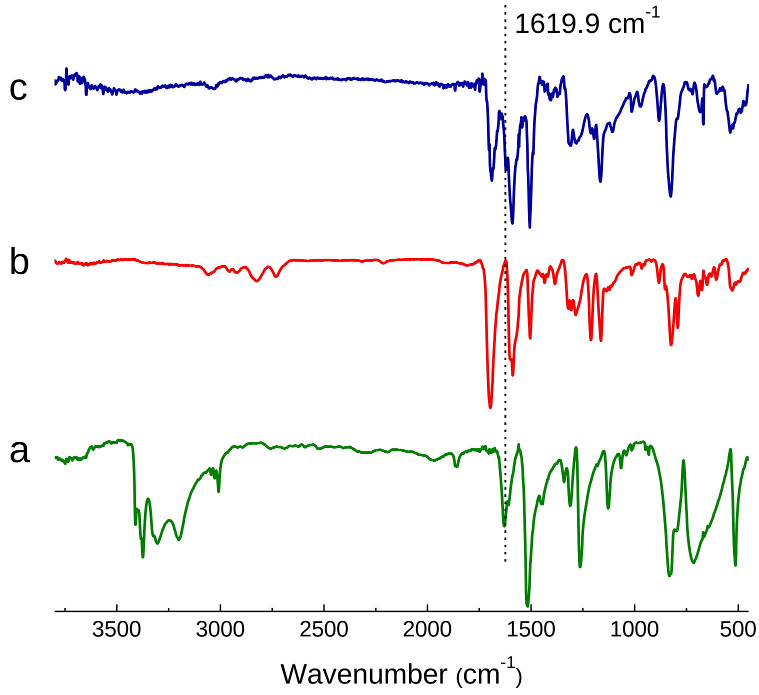

Supplementary Figure 4. FT-IR spectra of **a** DAB, **b** FPM, and **c** Tetra-COF-DAB.

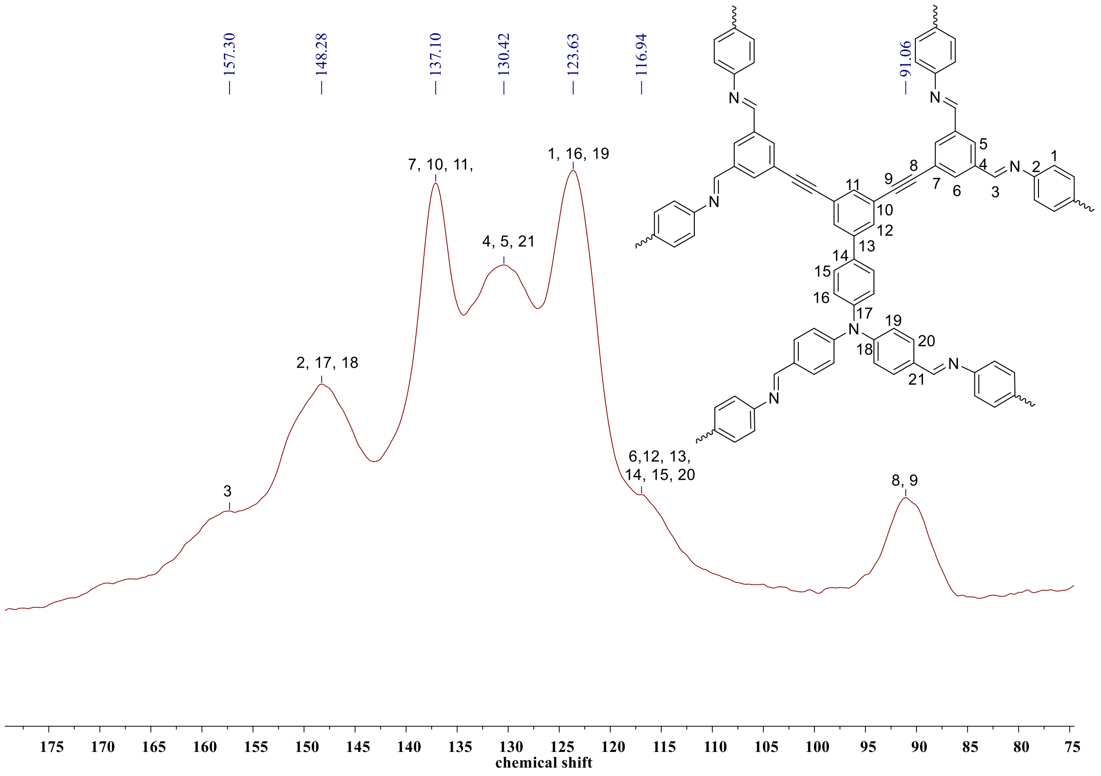

Supplementary Figure 5. Solid-state ^{13}C CP/MAS NMR spectrum of Tri-COF-DAB.

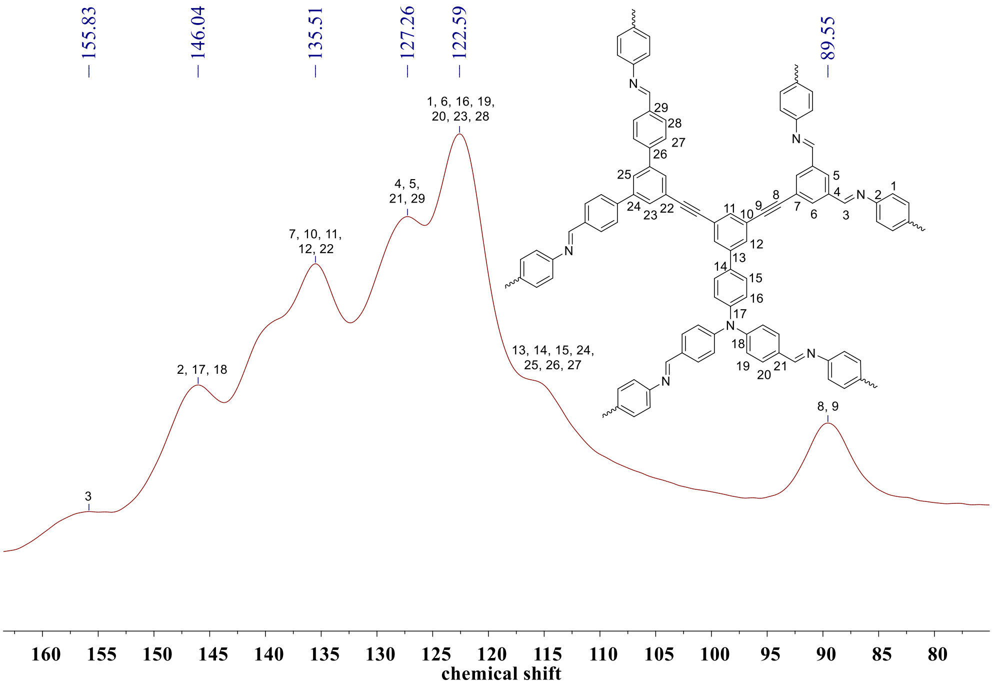

Supplementary Figure 6. Solid-state ^{13}C CP/MAS NMR spectrum of Tetra-COF-DAB.

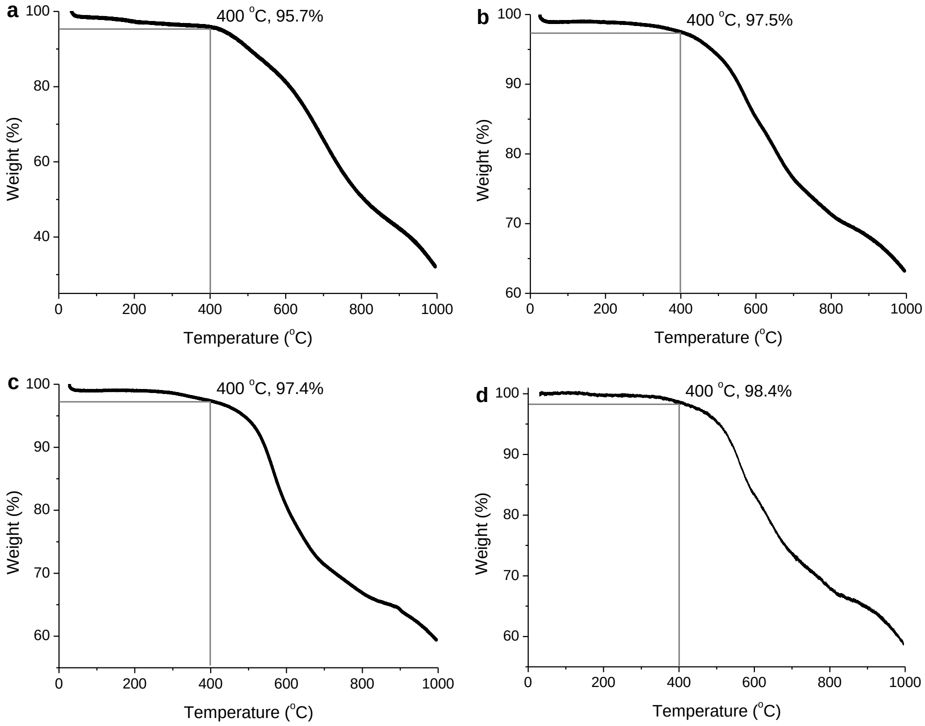

Supplementary Figure 7. TGA profiles of **a** Tri-COF-DAB, **b** Tetra-COF-DAB, **c** Tri-COF-BZ, and **d** Tetra-COF-BZ.

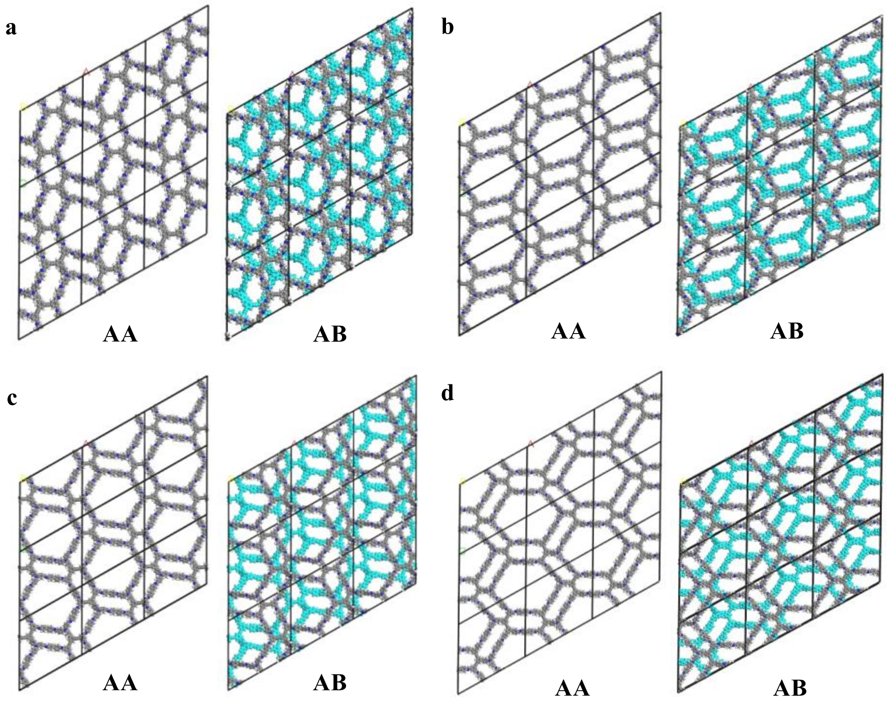

Supplementary Figure 8. Illustrations of AA and AB stacking models of **a** Tri-COF-DAB, **b** Tetra-COF-DAB, **c** Tri-COF-BZ and **d** Tetra-COF-BZ.

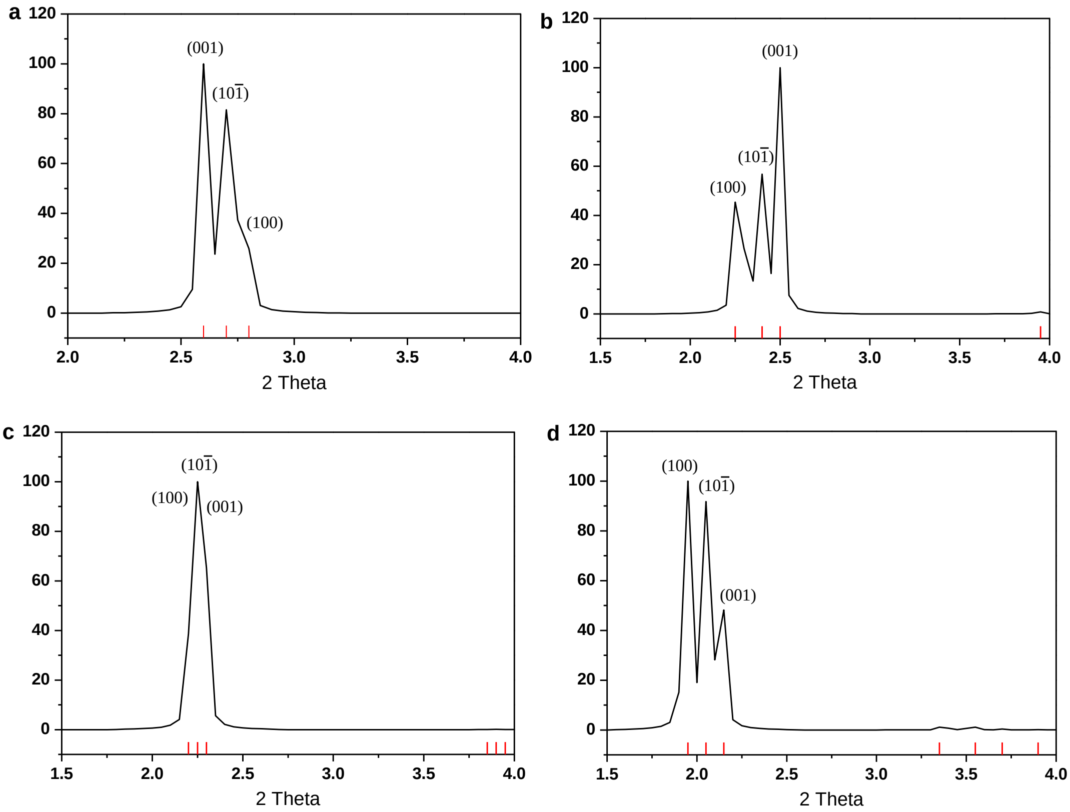

Supplementary Figure 9. The close-up views of the simulated PXRD patterns of **a** Tri-COF-DAB, **b** Tetra-COF-DAB, **c** Tri-COF-BZ, and **d** Tetra-COF-BZ with AA stacking models.

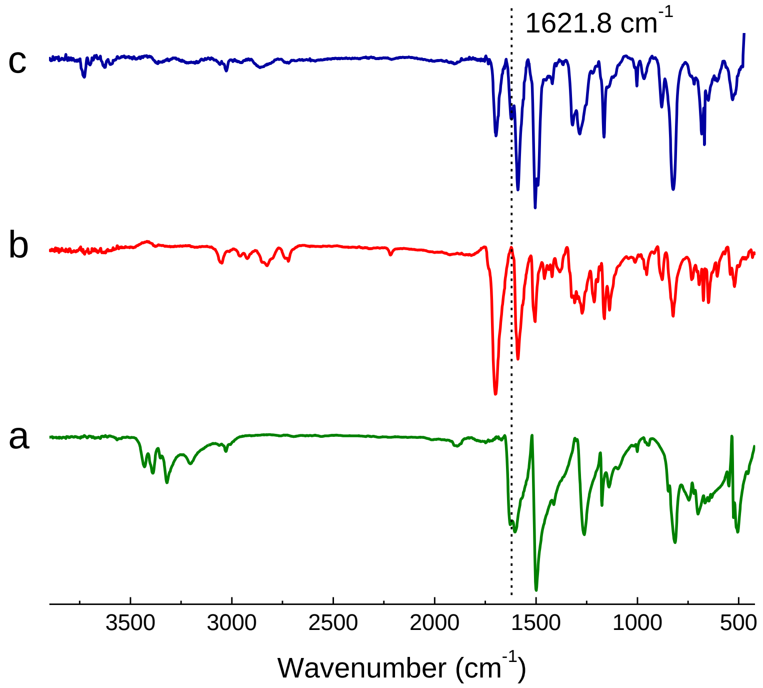

Supplementary Figure 10. FT-IR spectra of **a** BZ, **b** TPM, and **c** Tri-COF-BZ.

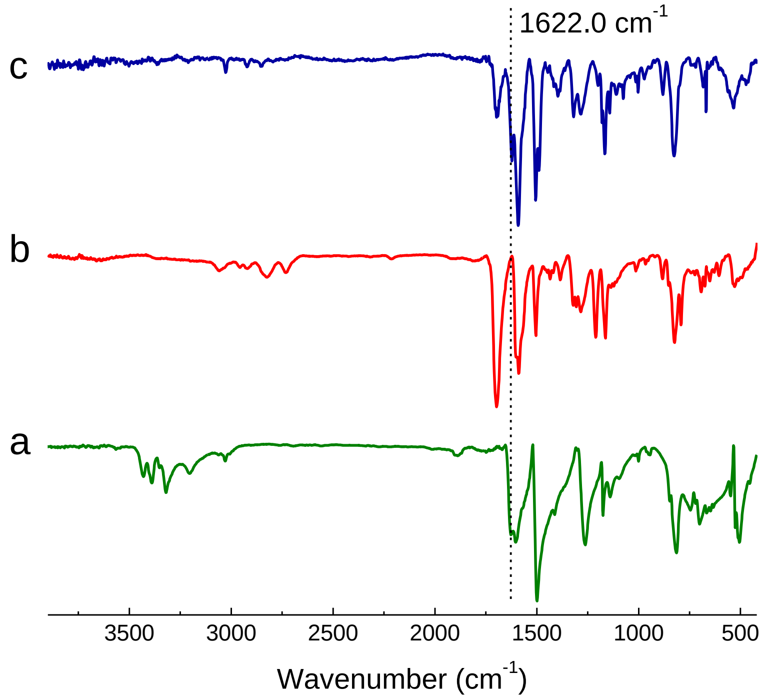

Supplementary Figure 11. FT-IR spectra of **a** BZ, **b** FPM, and **c** Tetra-COF-BZ.

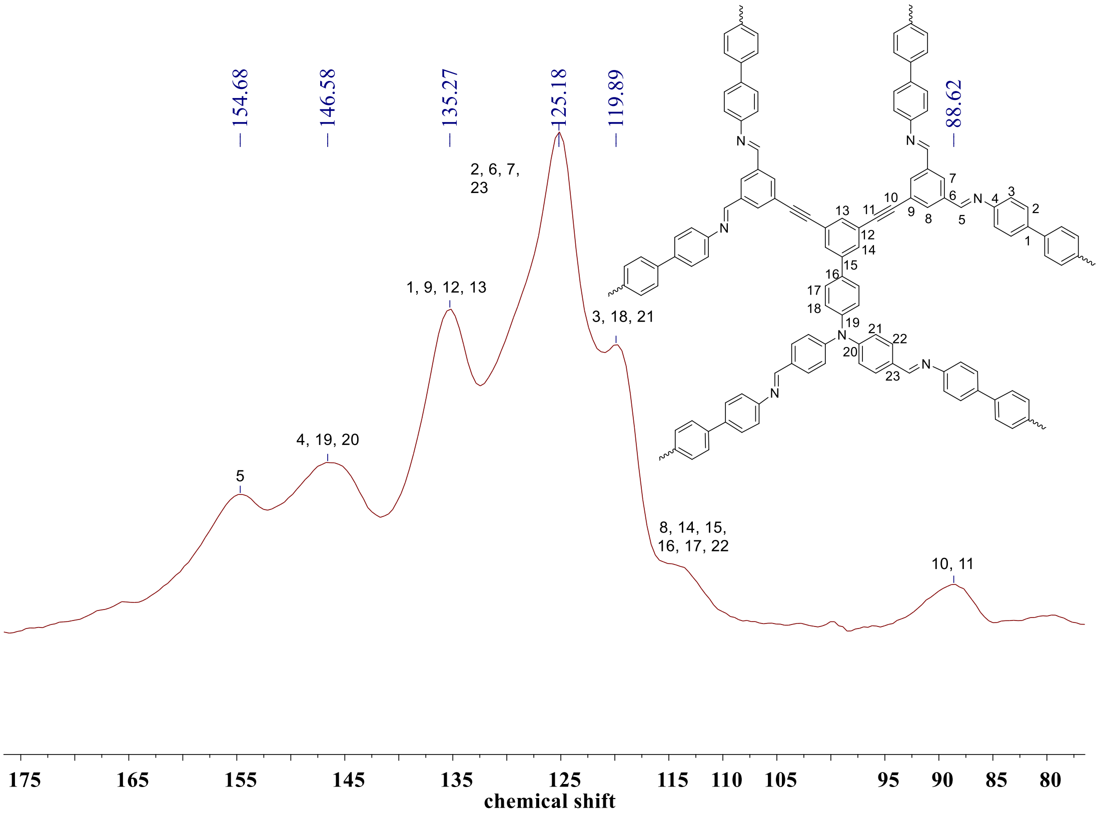

Supplementary Figure 12. Solid-state ^{13}C CP/MAS NMR Spectrum of Tri-COF-BZ.

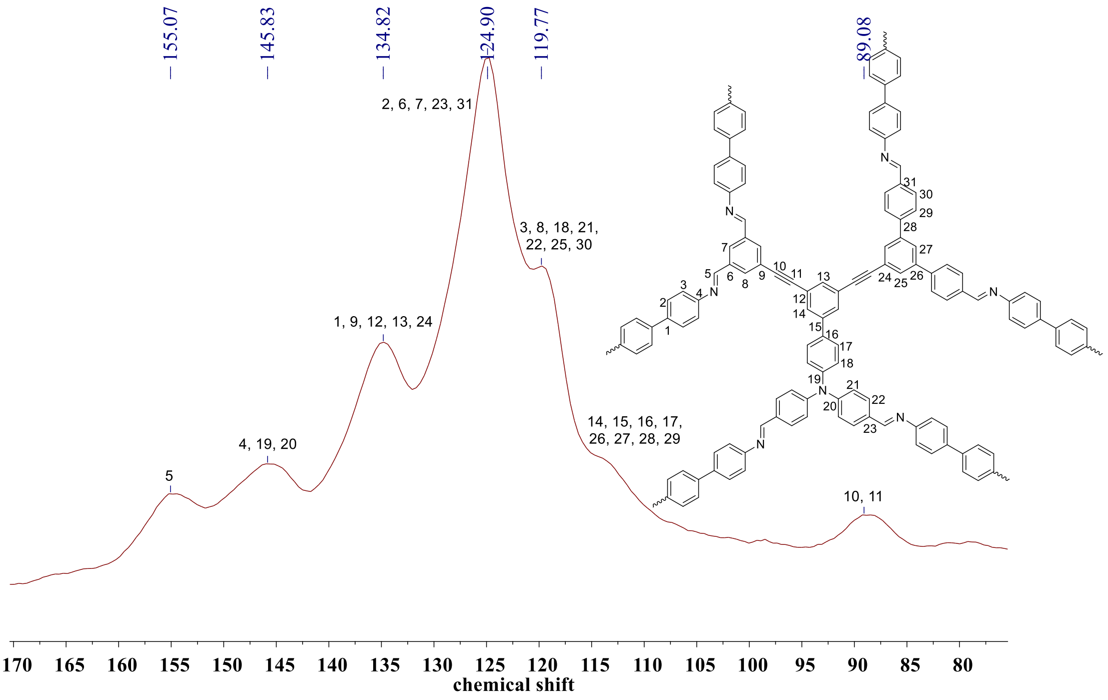

Supplementary Figure 13. Solid-state ^{13}C CP/MAS NMR Spectrum of Tetra-COF-BZ.

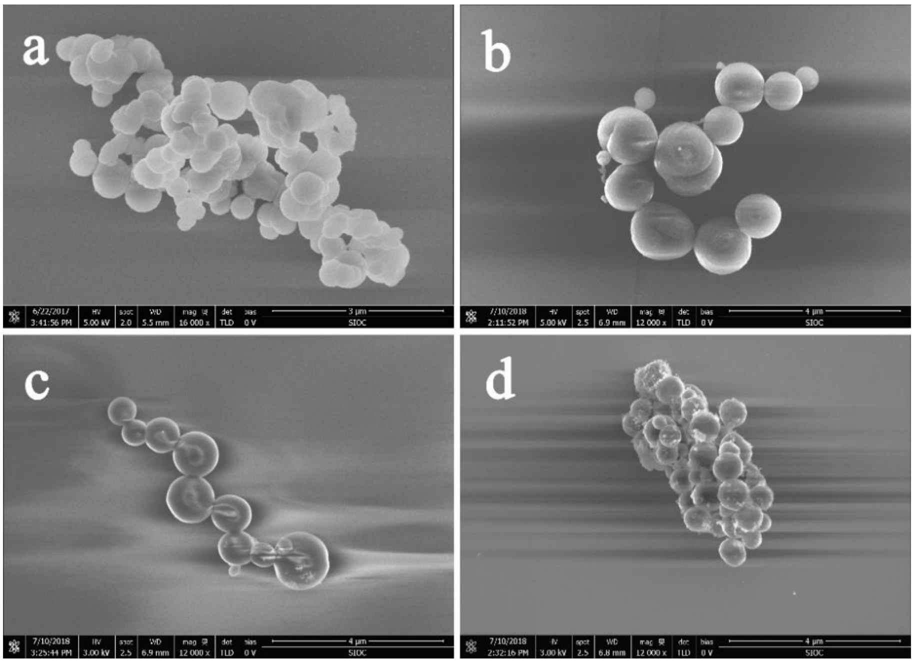

Supplementary Figure 14. SEM images of **a** Tri-COF-DAB, **b** Tetra-COF-DAB, **c** Tri-COF-BZ, and **d** Tetra-COF-BZ.

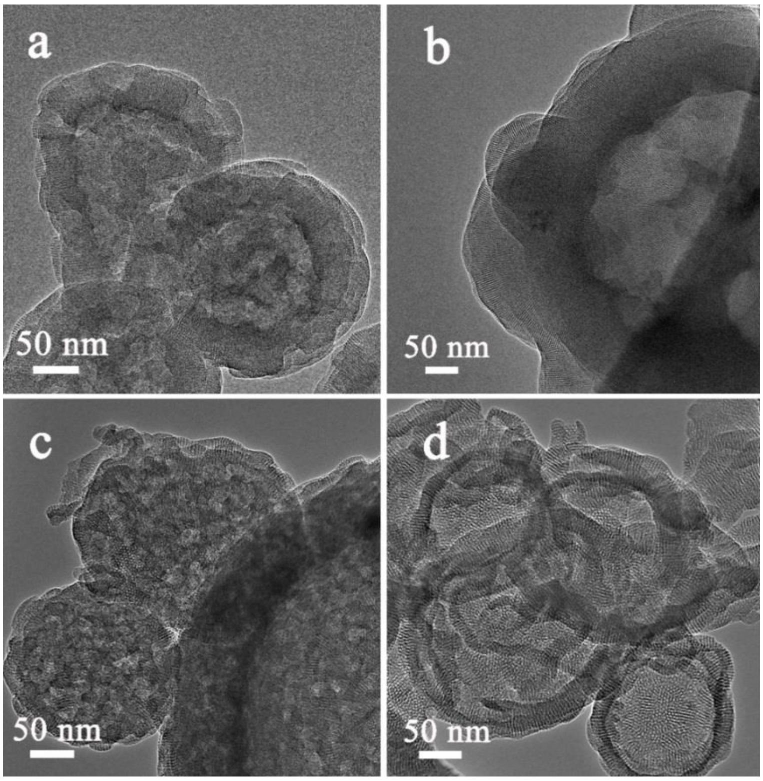

Supplementary Figure 15. TEM images of **a** Tri-COF-DAB, **b** Tetra-COF-DAB, **c** Tri-COF-BZ, and **d** Tetra-COF-BZ.

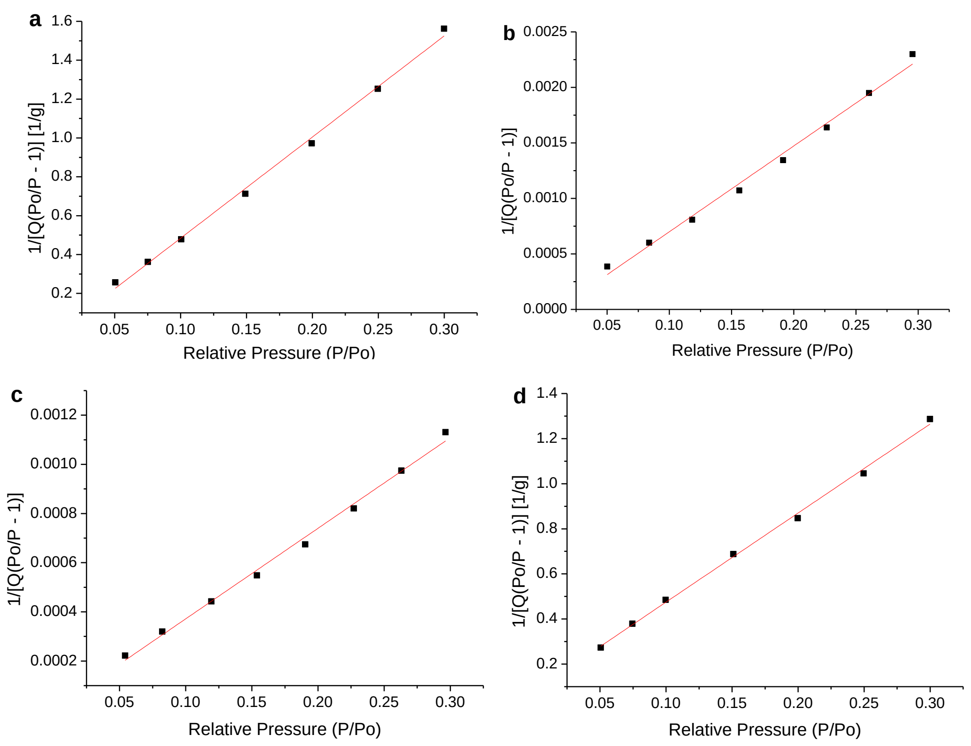

Supplementary Figure 16. BET surface area plots for **a** Tri-COF-DAB, **b** Tetra-COF-DAB, **c** Tri-COF-BZ, and **d** Tetra-COF-BZ.

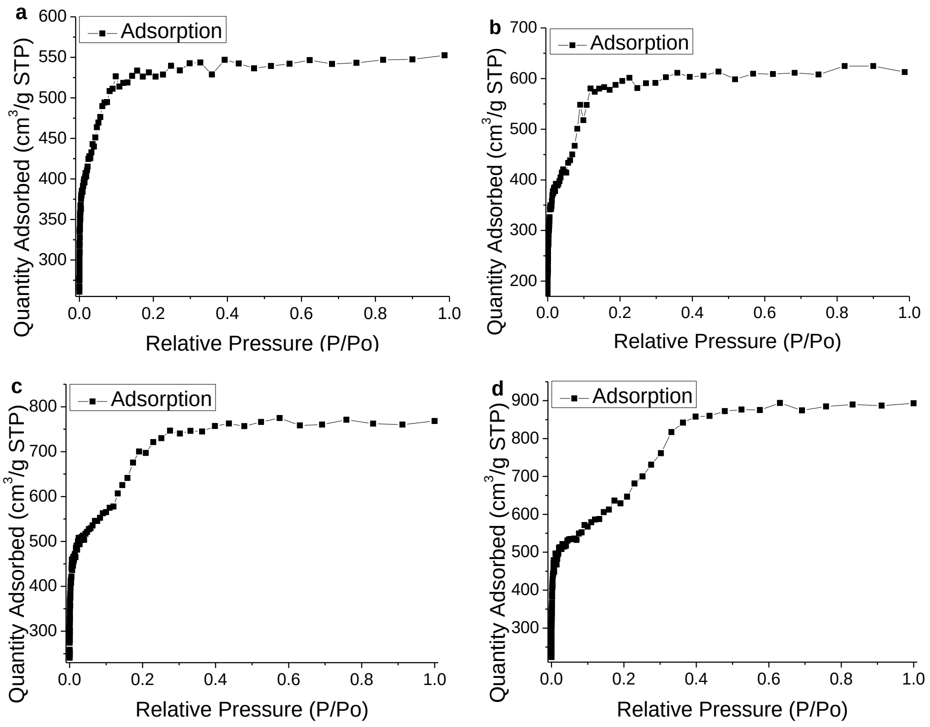

Supplementary Figure 17. Theoretical N_2 adsorption isotherms (77 K) of **a** Tri-COF-DAB, **b** Tetra-COF-DAB, **c** Tri-COF-BZ and **d** Tetra-COF-BZ.

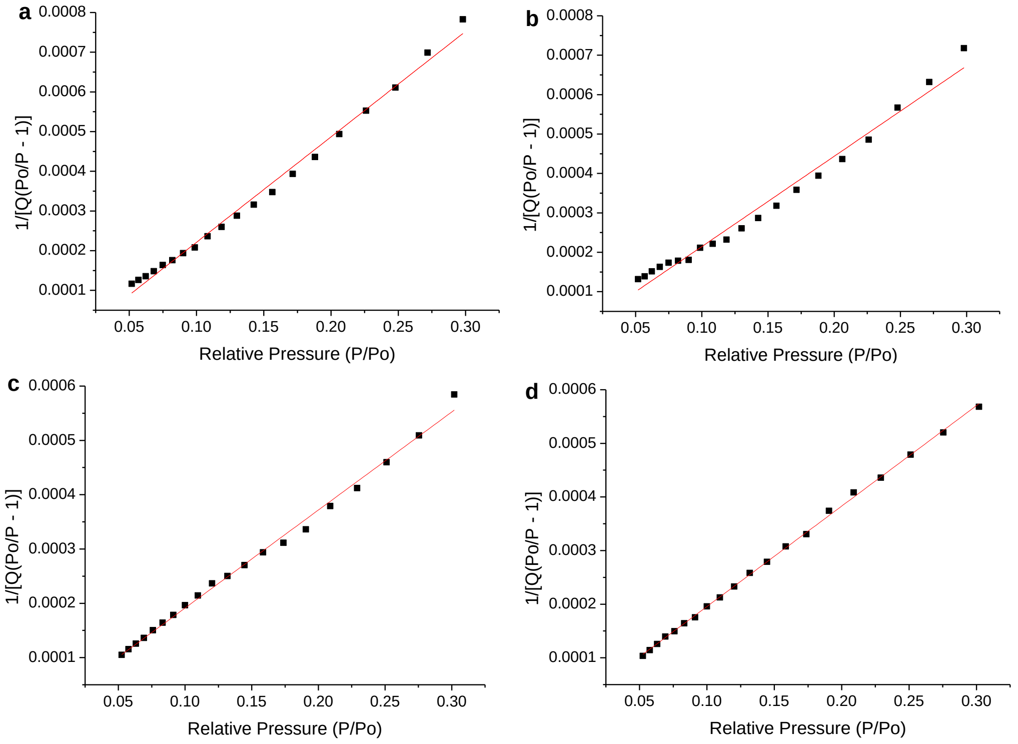

Supplementary Figure 18. Theoretical BET surface area plots for **a** Tri-COF-DAB, **b** Tetra-COF-DAB, **c** Tri-COF-BZ, and **d** Tetra-COF-BZ.

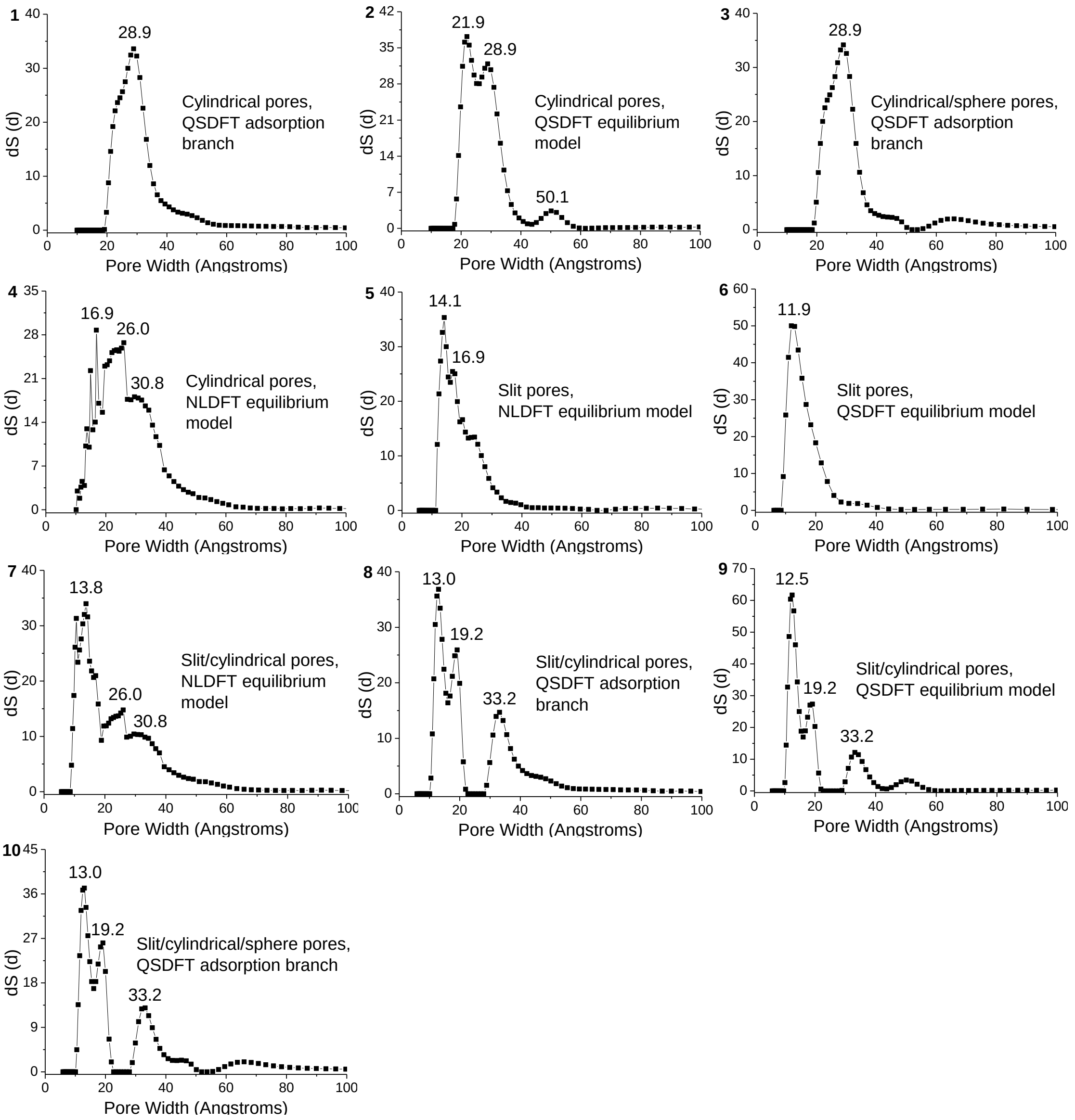

Supplementary Figure 19. (1-10) PSD profiles derived from different kernels/pore models, based on the nitrogen sorption isotherm of Tetra-COF-BZ at 77K.

Supplementary Table 1. Fractional atomic coordinates for the unit cell of Tri-COF-DAB with AA stacking.

*P*2							
a = 36.79 Å, *b* = 3.68 Å, and *c* = 39.23 Å, and $\alpha = \gamma = 90^o$ and $\beta = 120^o$							
N7	0.29395	0.48327	0.9289	C69	0.08934	0.29553	0.67482
C15	0.68271	0.4924	1.0283	N123	0.02728	0.43106	0.36913
C16	0.63948	0.41275	1.00599	N125	0.32605	0.61937	0.38393

C17	0.61839	0.42296	0.96468	C131	0.64429	0.65346	0.70882
C18	0.63951	0.50823	0.94424	C132	0.61391	0.7359	0.66948
C19	0.68282	0.58461	0.96636	C133	0.62421	0.72839	0.63954
C20	0.704	0.57505	1.00783	C134	0.66485	0.63915	0.64795
N21	0.61545	0.51705	0.90157	C135	0.69582	0.57915	0.68767
C22	0.62871	0.55795	0.87648	C136	0.68551	0.58629	0.7176
C23	0.59859	0.55747	0.83304	H151	0.6217	0.33654	1.01996
C24	0.55482	0.5412	0.81829	H152	0.58519	0.36087	0.94837
C25	0.5267	0.53458	0.7776	H153	0.70044	0.65474	0.95204
C26	0.54186	0.54097	0.75147	H154	0.73724	0.63546	1.02417
C27	0.58489	0.55878	0.76482	H155	0.66184	0.58577	0.88668
C28	0.61342	0.56996	0.80584	H156	0.54255	0.53345	0.83828
C29	0.2653	0.62356	0.59911	H157	0.51961	0.53312	0.7201
C30	0.24194	0.62592	0.55781	H158	0.64682	0.58148	0.81653
C31	0.25672	0.46473	0.53435	H159	0.25208	0.7549	0.61513
C32	0.29732	0.31092	0.55496	H160	0.21186	0.75261	0.54459
C33	0.3212	0.31319	0.59636	H161	0.31093	0.18255	0.53944
C34	0.30543	0.46669	0.6193	H162	0.35161	0.18264	0.61033
N35	0.2309	0.45251	0.49086	H163	0.17767	0.22354	0.51895
C36	0.25126	0.42716	0.46646	H164	0.10215	0.18116	0.4837
C37	0.18418	0.45102	0.47099	H165	0.0983	0.63361	0.38093
C38	0.16184	0.31527	0.48911	H166	0.17152	0.65757	0.41394
C39	0.11772	0.29246	0.46879	H167	0.20195	0.1201	0.42009
C40	0.09406	0.40233	0.42939	H168	0.22729	0.12167	0.37539
C41	0.11528	0.54582	0.41135	H169	0.33615	0.71877	0.45913
C42	0.15881	0.57033	0.43159	H170	0.30859	0.72412	0.50457
C43	0.23109	0.24828	0.42998	H171	0.0318	0.25832	0.42162
C44	0.24587	0.25309	0.4037	H172	0.27654	0.26214	0.3564
C45	0.28345	0.42462	0.41317	H173	0.3912	0.50961	0.66703

C46	0.30657	0.58602	0.45062	H174	0.39701	0.47736	0.7789
C47	0.29053	0.58854	0.47685	H175	0.27713	0.42842	0.6729
C48	0.04776	0.35824	0.40669	H176	0.1527	0.44503	0.79566
C49	0.29544	0.42739	0.38216	H177	0.57277	0.4808	0.70452
C50	0.33066	0.46705	0.66374	H178	0.08426	0.55017	0.75587
C51	0.37496	0.48816	0.68356	H179	0.00939	0.56651	0.70703
C52	0.39868	0.48918	0.72491	H180	0.03692	0.24092	0.6167
C53	0.37856	0.47404	0.74701	H181	0.11158	0.223	0.66552
C54	0.33486	0.45595	0.72838	H213	0.58222	0.80903	0.6617
C55	0.31093	0.45	0.68689	H214	0.60022	0.78757	0.60955
C56	0.31539	0.44735	0.75265	H215	0.72815	0.52894	0.69595
C57	0.44394	0.50629	0.74503	H216	0.70963	0.53235	0.74767
C58	0.30009	0.44349	0.77378	C8	0.30993	0.47694	0.90569
C59	0.48179	0.51933	0.76124	C9	0.28358	0.46071	0.86178
C60	0.16897	0.41978	0.77922	C10	0.23923	0.44716	0.84205
N61	0.1484	0.38255	0.74106	C11	0.21558	0.42936	0.80052
C62	0.10379	0.38044	0.71441	C12	0.23667	0.42605	0.77895
C63	0.59704	0.55558	0.73392	C13	0.28029	0.44121	0.7978
N64	0.63421	0.63941	0.74008	C14	0.30372	0.45808	0.8391
C65	0.07444	0.47653	0.72583	H147	0.34355	0.48917	0.91798
C66	0.03156	0.48787	0.69785	H148	0.22315	0.45018	0.85883
C67	0.01711	0.40461	0.65819	H149	0.21912	0.41455	0.74707
C68	0.04649	0.30631	0.64687	H150	0.33766	0.46978	0.85357

Supplementary Table 2. Fractional atomic coordinates for the unit cell of **Tetra-COF-DAB** with AA stacking.

*P*2							
a = 44.94 Å, b = 3.69 Å, and c = 40.89 Å, and $\alpha = \gamma = 90^{o}$ and $\beta = 120^{o}$							
C54	0.12512	0.48422	0.99213	H124	0.34123	0.79628	0.65787

C55	0.14401	0.42554	0.9736	H125	0.34471	0.24685	0.56704
C56	0.12761	0.42935	0.93423	H126	0.28392	0.19972	0.5338
C57	0.09229	0.49346	0.91251	H127	0.26055	0.74156	0.50487
C58	0.07357	0.56573	0.93099	H128	0.23407	0.72975	0.43794
C59	0.09	0.56128	0.97046	H129	0.14764	0.15915	0.43151
H111	0.17144	0.38115	0.98911	H130	0.17307	0.20135	0.49817
H112	0.14249	0.38	0.92048	N7	0.40258	0.50019	0.61704
H113	0.04649	0.62882	0.91547	N8	0.45357	0.51783	0.33119
H114	0.07525	0.61583	0.98426	C9	0.38592	0.57456	0.63471
C32	0.9678	0.468	0.19341	C10	0.43824	0.57454	0.35064
C33	0.94471	0.45447	0.20711	C11	0.40041	0.53667	0.33284
C34	0.95659	0.43205	0.24604	C12	0.38104	0.37916	0.29704
C35	0.99235	0.42457	0.27155	C13	0.34544	0.34616	0.28032
C42	0.9557	0.48431	0.15255	C14	0.32779	0.47968	0.29828
C43	0.93068	0.41875	0.25845	C15	0.34739	0.6314	0.33456
N44	0.93918	0.3839	0.29375	C16	0.38332	0.65701	0.35172
C45	0.91544	0.36823	0.30786	C17	0.17215	0.42664	0.38679
N46	0.92308	0.48557	0.12791	C18	0.14733	0.5202	0.61668
C60	0.88016	0.28958	0.28467	C19	0.19219	0.39876	0.36918
C61	0.85812	0.28657	0.29972	C20	0.17667	0.39084	0.32974
C62	0.87057	0.36165	0.33798	C21	0.14069	0.40055	0.30749
C63	0.90612	0.42403	0.36143	C22	0.1204	0.42376	0.32465
C64	0.92811	0.42768	0.3464	C23	0.13613	0.44212	0.36398
H99	0.9173	0.45956	0.1874	C24	0.19782	0.38362	0.31222
H104	0.97468	0.48406	0.14369	C25	0.08332	0.43011	0.30211
H105	0.90398	0.44759	0.23695	N26	0.15321	0.36344	0.64787
H115	0.86939	0.22576	0.25511	C27	0.05234	0.43446	0.2829
H116	0.83112	0.22797	0.28138	C28	0.21523	0.38605	0.29723
H117	0.91733	0.46598	0.39145	C29	0.01555	0.44035	0.25789

H118	0.95522	0.48028	0.36489	C30	0.23476	0.39934	0.27783
C1	0.48999	0.53351	0.34498	C31	0.00313	0.46155	0.21905
C2	0.51456	0.49304	0.38344	C36	0.27085	0.42961	0.29792
C3	0.54964	0.48803	0.39518	C37	0.28927	0.46383	0.27841
C4	0.56104	0.5244	0.3691	C38	0.27053	0.47724	0.23874
C5	0.53652	0.56479	0.3306	C39	0.23488	0.42137	0.21835
C6	0.50136	0.56728	0.3188	C40	0.2171	0.38671	0.23828
C65	0.20478	0.35632	0.62208	C41	0.21699	0.38997	0.17641
C66	0.22609	0.3472	0.60586	C47	0.23402	0.23019	0.15905
C67	0.21509	0.48793	0.56929	C48	0.21839	0.21061	0.11988
C68	0.18169	0.63605	0.55032	C49	0.18519	0.34652	0.09691
C69	0.16052	0.6438	0.56608	C50	0.16756	0.49892	0.1139
C70	0.17159	0.5045	0.60203	C51	0.18335	0.52072	0.15333
N71	0.23661	0.47624	0.55125	C52	0.17009	0.32718	0.05557
C72	0.22009	0.46847	0.50949	N53	0.14081	0.47843	0.03262
C73	0.27454	0.47872	0.5743	H88	0.39912	0.68099	0.66315
C74	0.29245	0.64232	0.61038	H89	0.45275	0.66008	0.37983
C75	0.32858	0.66341	0.63062	H90	0.39344	0.2754	0.28208
C76	0.34802	0.52947	0.61505	H91	0.33187	0.21037	0.25331
C77	0.33072	0.35879	0.57989	H92	0.33503	0.74058	0.34931
C78	0.29506	0.32832	0.56058	H93	0.39769	0.77956	0.3794
C79	0.23588	0.61881	0.49016	H94	0.12386	0.6719	0.60015
C80	0.22073	0.60439	0.45088	H95	0.21991	0.38121	0.386
C81	0.1886	0.44113	0.42875	H96	0.12856	0.39217	0.27697
C82	0.17222	0.29384	0.44722	H97	0.12009	0.47304	0.37663
C83	0.18763	0.30844	0.48649	H98	0.02113	0.47219	0.20872
H84	0.50689	0.45426	0.40438	H101	0.28432	0.42258	0.32847
H85	0.56813	0.45092	0.42484	H102	0.28379	0.53307	0.22335
H86	0.54416	0.58596	0.30947	H103	0.18953	0.34612	0.22318

H87	0.4828	0.59296	0.28902	H106	0.25942	0.11483	0.17573
H119	0.21417	0.24515	0.65004	H107	0.23232	0.08751	0.10747
H120	0.25107	0.2291	0.62233	H108	0.14188	0.60643	0.09678
H121	0.17057	0.73511	0.52228	H109	0.16961	0.64965	0.16561
H122	0.135	0.75831	0.54968	H110	0.18423	0.18095	0.04479
H123	0.27894	0.76082	0.6231	H100	1.00207	0.40772	0.30166

Supplementary Table 3. Fractional atomic coordinates for the unit cell of **Tri-COF-BZ** with AA stacking.

*P*2							
a = 45.95 Å, b = 3.85 Å, and c = 44.59 Å, and $\alpha = \gamma = 90^{o}$ and $\beta = 120^{o}$							
C2	0.16552	0.47069	0.01532	N77	0.26732	0.51114	0.49669
C3	0.18531	0.4565	0.05139	C78	0.30409	0.51147	0.51216
C4	0.17199	0.33086	0.07148	C79	0.24558	0.50826	0.45883
C5	0.13877	0.20448	0.05472	C80	0.21365	0.34991	0.44279
C6	0.11896	0.21966	0.01869	C81	0.1926	0.3526	0.40691
N7	0.19282	0.3379	0.10857	C82	0.20282	0.51183	0.38546
C8	0.18186	0.32627	0.13036	C83	0.23469	0.66663	0.40086
C9	0.20502	0.34664	0.16809	C84	0.25555	0.66514	0.43682
C10	0.24016	0.35894	0.18251	C85	0.3199	0.35339	0.4955
C11	0.2614	0.39019	0.2185	C86	0.35488	0.34208	0.51082
C12	0.24718	0.407	0.24	C87	0.3754	0.48614	0.54359
C13	0.21237	0.39297	0.22581	C88	0.36025	0.65099	0.56029
C14	0.1914	0.36225	0.19005	C89	0.32529	0.66637	0.54462
C24	0.00056	0.59379	0.22105	H125	0.46959	0.75121	0.3075
C25	0.02283	0.63441	0.25658	H126	0.531	0.76926	0.3437
C26	0.01044	0.68398	0.27922	H127	0.52339	0.34856	0.42928
C29	0.41229	0.4564	0.56009	H128	0.46276	0.32459	0.39323
C30	0.21005	0.54336	0.58659	H129	0.18204	0.7892	0.52013

C31	0.18035	0.516	0.34725	H130	0.20588	0.77129	0.48296
C32	0.14541	0.55897	0.33267	H131	0.29322	0.24926	0.56284
C33	0.12423	0.55843	0.29668	H132	0.26924	0.26636	0.60002
C34	0.13775	0.51962	0.27491	H133	0.20425	0.22951	0.4578
C35	0.17242	0.47913	0.28909	H134	0.16846	0.22417	0.39598
C36	0.19361	0.4753	0.32506	H135	0.24333	0.79619	0.3852
C37	0.18635	0.44422	0.26671	H136	0.27969	0.78402	0.44706
C38	0.08848	0.59117	0.28211	H137	0.30551	0.24009	0.47038
C39	0.19814	0.41873	0.24801	H138	0.36591	0.21496	0.4972
C40	0.05853	0.61353	0.27019	H139	0.37535	0.76805	0.58556
C41	0.29823	0.40827	0.23338	H140	0.31516	0.79143	0.55878
N42	0.31762	0.46613	0.26629	C1	0.13198	0.35665	-0.00137
N45	0.43173	0.5745	0.59121	C15	0.11044	0.38752	-0.03947
N46	0.17911	0.63629	0.57533	C16	0.07717	0.50651	-0.05411
C59	0.42433	0.52328	0.32582	C17	0.05714	0.54504	-0.08994
C61	0.40401	0.6296	0.33939	C18	0.06991	0.46844	-0.11182
C62	0.36906	0.61146	0.31923	C19	0.10296	0.34063	-0.09733
C63	0.35364	0.48883	0.28516	C20	0.12304	0.30239	-0.06147
C64	0.37388	0.38221	0.27137	H98	0.06686	0.57668	-0.03778
C65	0.40892	0.40091	0.29155	H99	0.0317	0.64108	-0.1007
H90	0.17615	0.58026	0.00055	H100	0.11326	0.26457	-0.11346
H91	0.21087	0.55261	0.06384	H101	0.14829	0.20183	-0.05094
H92	0.128	0.0906	0.06912	N21	-0.04844	0.51754	0.14845
H93	0.09348	0.1214	0.00644	C22	-0.05857	0.55644	0.17073
H94	0.15517	0.31991	0.1212	C23	-0.03434	0.60203	0.20798
H95	0.25099	0.34754	0.16582	C27	-0.02427	0.69029	0.26664
H96	0.26308	0.43229	0.26781	C28	-0.04653	0.65042	0.23093
H97	0.16438	0.35449	0.17927	C43	-0.03672	0.72843	0.29122
H103	0.01046	0.5531	0.20378	N44	-0.06856	0.69842	0.28072

H104	0.02785	0.71212	0.30674	C47	-0.0839	0.71717	0.30192
H106	0.42252	0.325	0.54601	C48	-0.06749	0.85916	0.33552
H107	0.22588	0.46403	0.61329	C49	-0.08333	0.86107	0.35533
H108	0.13464	0.5955	0.34921	C50	-0.11581	0.72324	0.34198
H109	0.12135	0.52029	0.24704	C51	-0.13236	0.58937	0.30813
H110	0.22035	0.43646	0.33566	C52	-0.11656	0.58847	0.28833
H111	0.30849	0.38016	0.21635	H102	-0.08512	0.56544	0.16225
H121	0.41516	0.72955	0.36553	H105	-0.07341	0.65463	0.22092
H122	0.35383	0.69392	0.3302	H112	-0.01859	0.76348	0.31838
H123	0.36303	0.27745	0.24551	H113	-0.04277	0.9734	0.34648
H124	0.42402	0.31162	0.28067	H114	-0.07031	0.97449	0.38098
C60	0.46145	0.53571	0.34756	H115	-0.1572	0.47682	0.29727
C66	0.48122	0.65921	0.33396	H116	-0.12953	0.47923	0.2625
C67	0.5162	0.67136	0.3546	C53	-0.16385	0.66473	0.4035
C68	0.53217	0.55854	0.38906	C54	-0.13221	0.713	0.36339
C69	0.51237	0.43594	0.40282	C55	-0.18247	0.7576	0.36835
C70	0.47738	0.4248	0.3822	C56	-0.16676	0.7815	0.34851
C71	0.20644	0.66979	0.52966	C57	-0.11359	0.62803	0.39875
C72	0.22055	0.66337	0.50827	C58	-0.12928	0.6074	0.41863
C73	0.2523	0.513	0.51911	H117	-0.20898	0.81699	0.35632
C74	0.26899	0.3661	0.5526	H118	-0.18159	0.85667	0.32158
C75	0.25527	0.3779	0.57434	H119	-0.08702	0.57031	0.41089
C76	0.22387	0.52959	0.56306	H120	-0.11461	0.53884	0.44576

Supplementary Table 4. Fractional atomic coordinates for the unit cell of **Tetra-COF-BZ** with AA stacking.

*P*2							
a = 52.50 Å, b = 3.85 Å, and c = 47.86 Å, and $\alpha = \gamma = 90^{o}$ and $\beta = 120^{o}$							
C1	0.39711	0.38977	0.38327	C81	0.12101	0.42475	0.42406

C2	0.58334	0.36438	0.63082	C82	0.13217	0.58061	0.45456
C3	0.5539	0.47638	0.61339	C83	0.16257	0.5935	0.47606
C4	0.53609	0.46864	0.62748	C84	0.08906	0.40697	0.40065
C5	0.54751	0.35576	0.65933	N85	0.06989	0.5486	0.4068
C6	0.57655	0.23131	0.67638	C86	0.03837	0.55015	0.38606
C7	0.59423	0.23515	0.66225	C87	0.02424	0.44497	0.35346
C8	0.36745	0.48653	0.36385	C88	0.9935	0.4485	0.33466
C9	0.34893	0.50978	0.37689	C89	0.97621	0.55691	0.3479
C10	0.35971	0.44479	0.40964	C90	0.99038	0.66737	0.38022
C11	0.3893	0.34622	0.42921	C91	0.02108	0.66514	0.39895
C12	0.4078	0.31876	0.41611	C92	0.10379	0.42056	0.6762
N13	0.52946	0.37236	0.67402	C93	0.07308	0.42508	0.65817
N14	0.33981	0.46621	0.42193	C94	0.05646	0.5502	0.67166
C15	0.53989	0.45157	0.7042	C95	0.07131	0.66613	0.70384
C16	0.52115	0.46064	0.71906	C96	0.10212	0.66285	0.72188
C17	0.34728	0.5515	0.4513	C97	0.3009	0.56969	0.89628
C18	0.3254	0.55372	0.46209	C98	0.32145	0.56694	0.9294
C19	0.29744	0.40425	0.44336	C99	0.35068	0.46177	0.94065
C20	0.27766	0.39287	0.45487	C100	0.35882	0.35686	0.91811
C21	0.28523	0.53513	0.48515	C101	0.33835	0.35945	0.88515
C22	0.31296	0.6941	0.50331	H102	0.54488	0.58012	0.58925
C23	0.33289	0.69984	0.49204	H103	0.5137	0.56302	0.61398
C24	0.49286	0.31671	0.70409	H104	0.58552	0.12968	0.70059
C25	0.47651	0.3151	0.71995	H105	0.61646	0.136	0.67594
C26	0.48761	0.4619	0.751	H106	0.3586	0.54863	0.3386
C27	0.51579	0.61388	0.76493	H107	0.32616	0.58381	0.36151
C28	0.53237	0.60953	0.74956	H108	0.39795	0.28228	0.45438
N29	0.47092	0.45397	0.76825	H109	0.43031	0.23582	0.43155
C30	0.48675	0.45001	0.80382	H110	0.56279	0.52537	0.71895

C31	0.43858	0.45379	0.74993	H111	0.36965	0.63048	0.46815
C32	0.42278	0.61532	0.7195	H112	0.29117	0.2863	0.42027
C33	0.39206	0.6082	0.70159	H113	0.25687	0.26128	0.44048
C34	0.37573	0.44224	0.71362	H114	0.31932	0.81637	0.52624
C35	0.39111	0.28498	0.74413	H115	0.35419	0.82046	0.5067
C36	0.42188	0.2903	0.76181	H116	0.48359	0.19874	0.68045
C37	0.47492	0.60225	0.82173	H117	0.45507	0.20098	0.70741
C38	0.49098	0.61752	0.85553	H118	0.52597	0.72543	0.78845
C39	0.51954	0.48214	0.87264	H119	0.55416	0.72337	0.76168
C40	0.53121	0.31977	0.85535	H120	0.434	0.74483	0.70902
C41	0.515	0.30145	0.82177	H121	0.38112	0.74038	0.67855
C42	0.34292	0.43549	0.69454	H122	0.37943	0.14956	0.75415
C43	0.53714	0.51843	0.90816	H123	0.43241	0.16706	0.78494
C44	0.32799	0.40998	0.66059	H124	0.45323	0.71043	0.80996
C45	0.29715	0.41199	0.64267	H125	0.48126	0.74205	0.86827
C46	0.28093	0.43385	0.65851	H126	0.5531	0.20901	0.86771
C47	0.2954	0.45652	0.69216	H127	0.52559	0.18456	0.81009
C48	0.3262	0.45939	0.71008	H128	0.52702	0.63827	0.92063
C49	0.28216	0.40652	0.60791	H129	0.34025	0.38777	0.64799
C50	0.27853	0.47964	0.70827	H130	0.25709	0.43627	0.64467
N51	0.56445	0.41726	0.92368	H131	0.33703	0.48627	0.73607
C52	0.58496	0.4382	0.95779	H132	0.55559	0.66165	0.97282
C53	0.26433	0.49726	0.7217	H133	0.59217	0.68177	0.02987
C54	0.27021	0.41202	0.57884	H135	0.6199	0.21985	0.95209
C55	0.2475	0.51595	0.73789	H136	0.20594	0.53711	0.69393
C56	0.25745	0.43161	0.54431	H137	0.20228	0.55324	0.782
C57	0.57744	0.56792	0.98024	H138	0.28568	0.49629	0.78489
C58	0.59844	0.57809	0.01313	H139	0.29973	0.45102	0.54795
C59	0.62731	0.45995	0.02431	H140	0.22518	0.55699	0.45231

C60	0.63477	0.33333	1.00182	H141	0.21264	0.39389	0.53373
C61	0.61376	0.32215	0.96898	H142	0.15869	0.58026	0.68944
C62	0.21686	0.53497	0.72006	H143	0.24909	0.55006	0.83591
C63	0.20022	0.54763	0.73563	H144	0.18557	0.17119	0.42999
C64	0.21485	0.54344	0.76967	H145	0.13278	0.15569	0.39246
C65	0.24581	0.52489	0.7878	H146	0.11747	0.69546	0.46169
C66	0.26187	0.51095	0.77151	H147	0.1705	0.72248	0.49904
C67	0.2762	0.46286	0.53163	H148	0.08208	0.26764	0.37837
C68	0.26489	0.50513	0.49834	H149	0.03655	0.36079	0.34216
C69	0.23418	0.51117	0.47785	H150	0.9832	0.36002	0.3099
C70	0.2149	0.4652	0.49007	H151	0.97768	0.75838	0.39094
C71	0.2269	0.42882	0.52365	H152	0.03149	0.75169	0.42378
C72	0.18246	0.45054	0.46756	H153	0.11564	0.31202	0.66518
C73	0.16778	0.56022	0.71527	H154	0.06226	0.32439	0.63378
C74	0.26147	0.5196	0.82356	H155	0.05911	0.76555	0.71485
N75	0.28981	0.47305	0.83955	H156	0.11314	0.75719	0.74649
C76	0.3093	0.46484	0.87387	H157	0.27885	0.65983	0.88848
N77	0.15039	0.55041	0.7273	H158	0.31466	0.65391	0.94621
C78	0.1187	0.54623	0.70815	H159	0.381	0.26906	0.92601
C79	0.17108	0.29242	0.4371	H160	0.34515	0.27837	0.8682
C80	0.14079	0.28067	0.41565	H134	0.65668	0.23569	1.00971

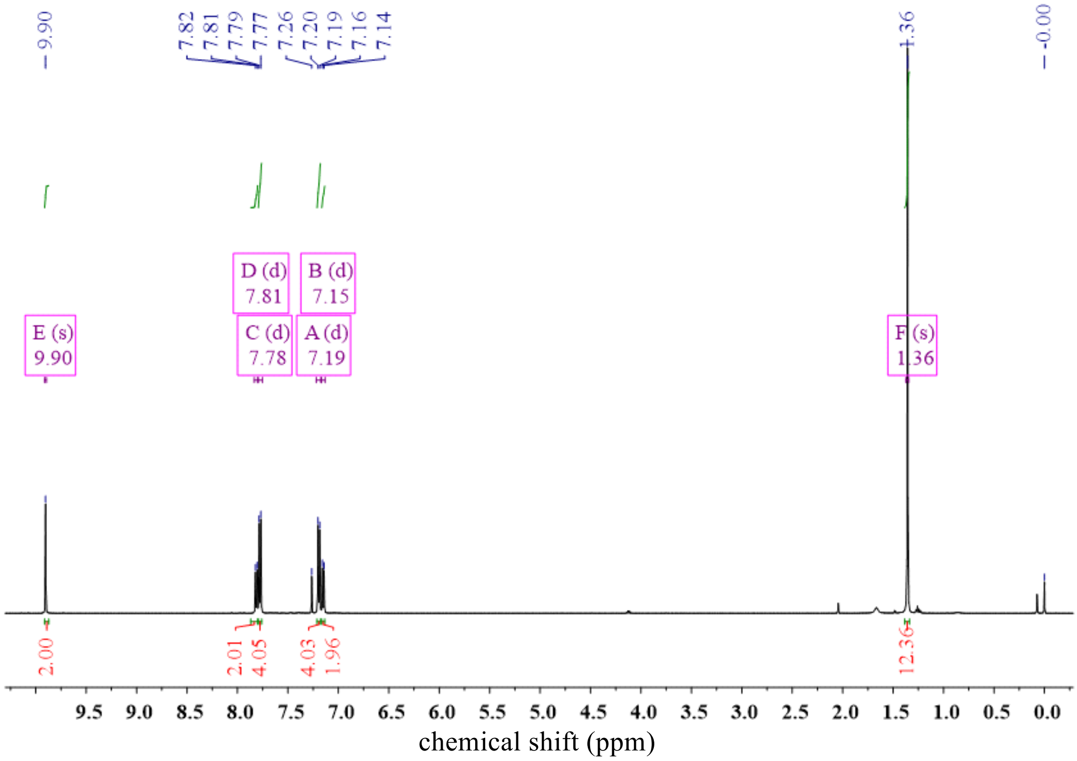

Supplementary Figure 20. ^{1}H NMR (500 MHz, $CDCl_3$) spectrum of 1c.

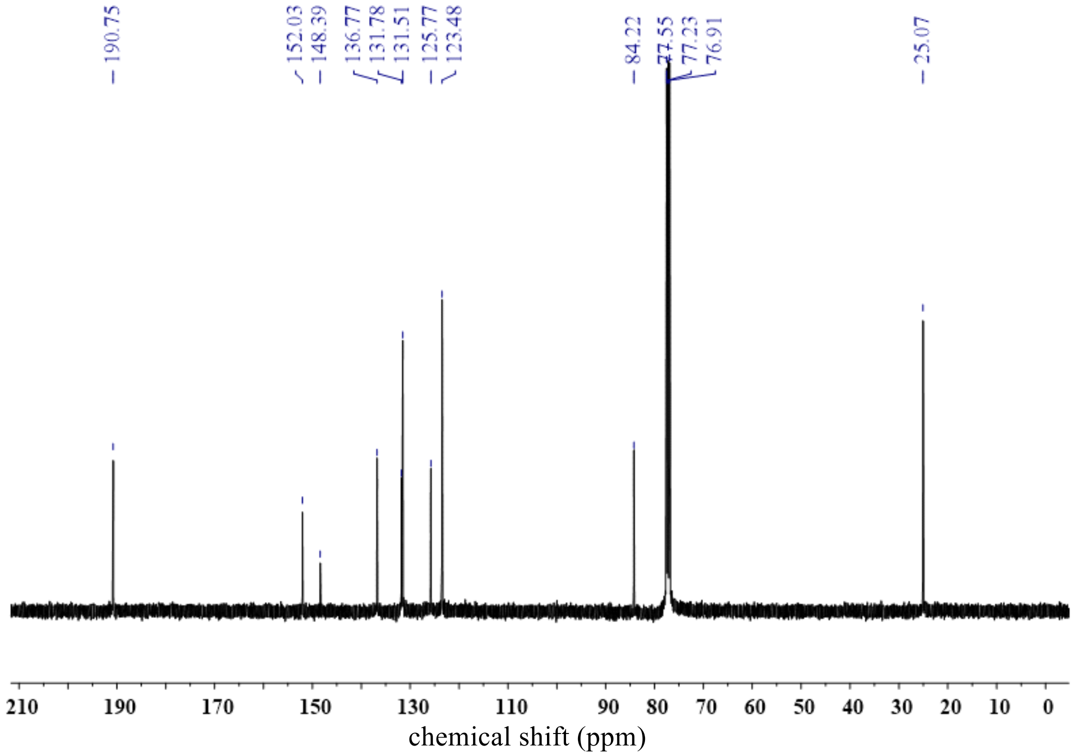

Supplementary Figure 21. ^{13}C NMR (101 MHz, $CDCl_3$) spectrum of 1c.

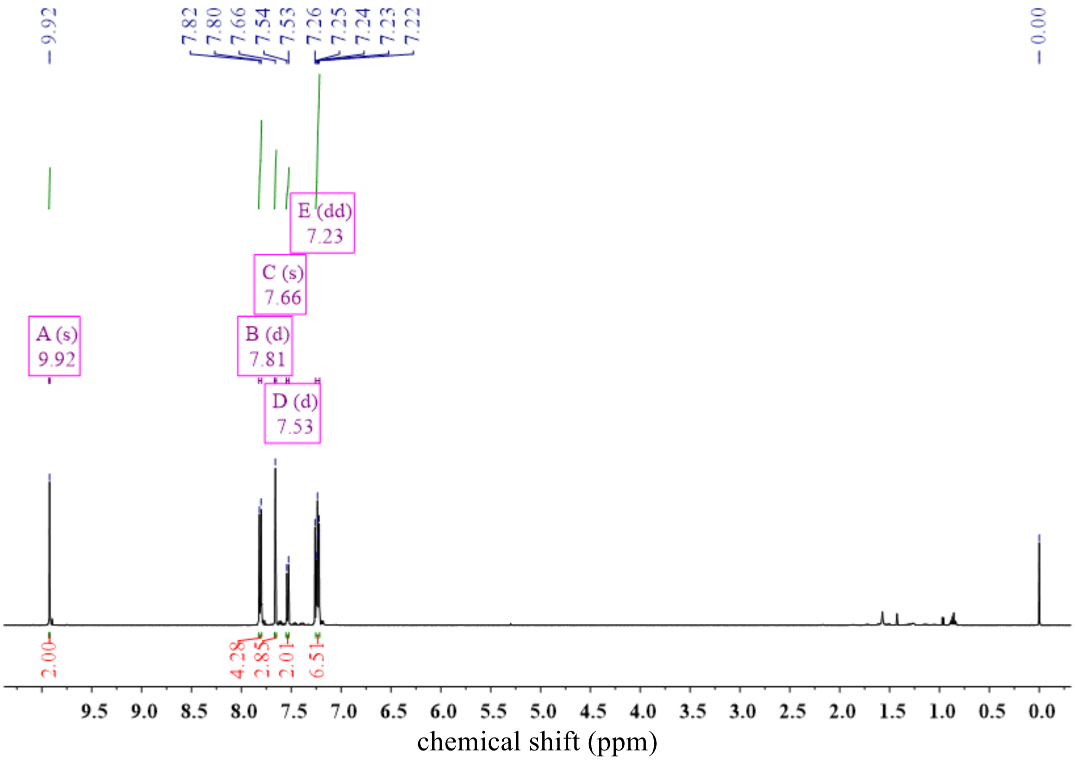

Supplementary Figure 22. ^{1}H NMR (500 MHz, $CDCl_3$) spectrum of 1d.

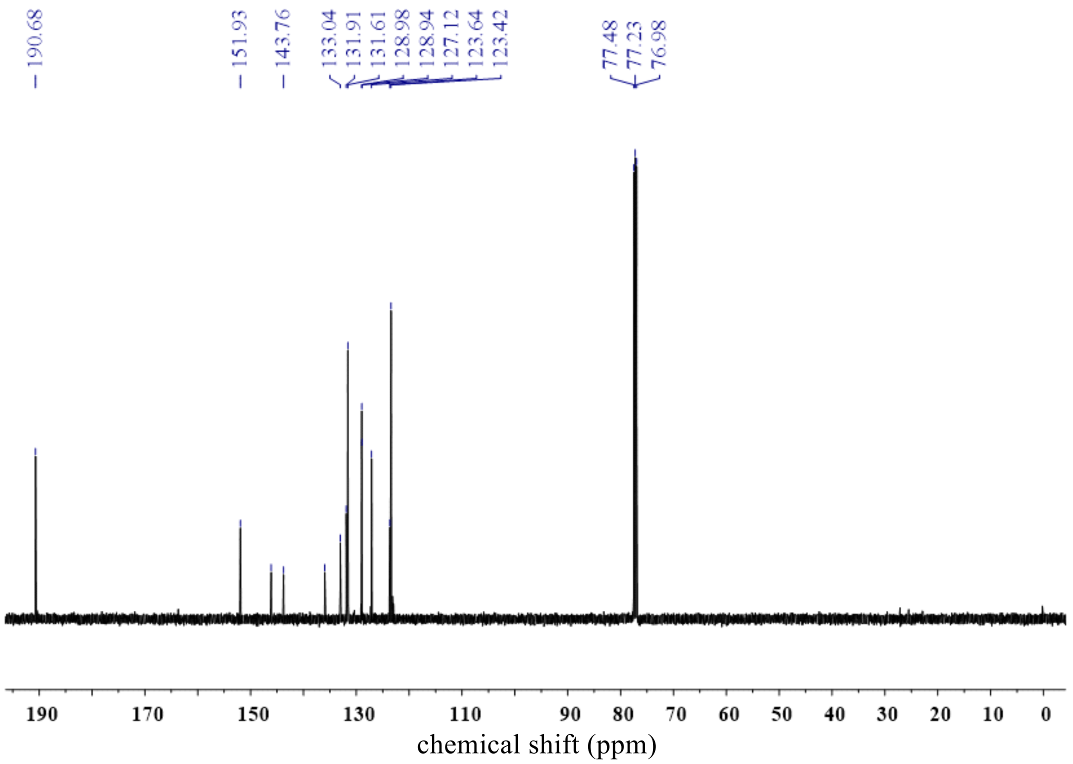

Supplementary Figure 23. ^{13}C NMR (126 MHz, $CDCl_3$) spectrum of 1d.

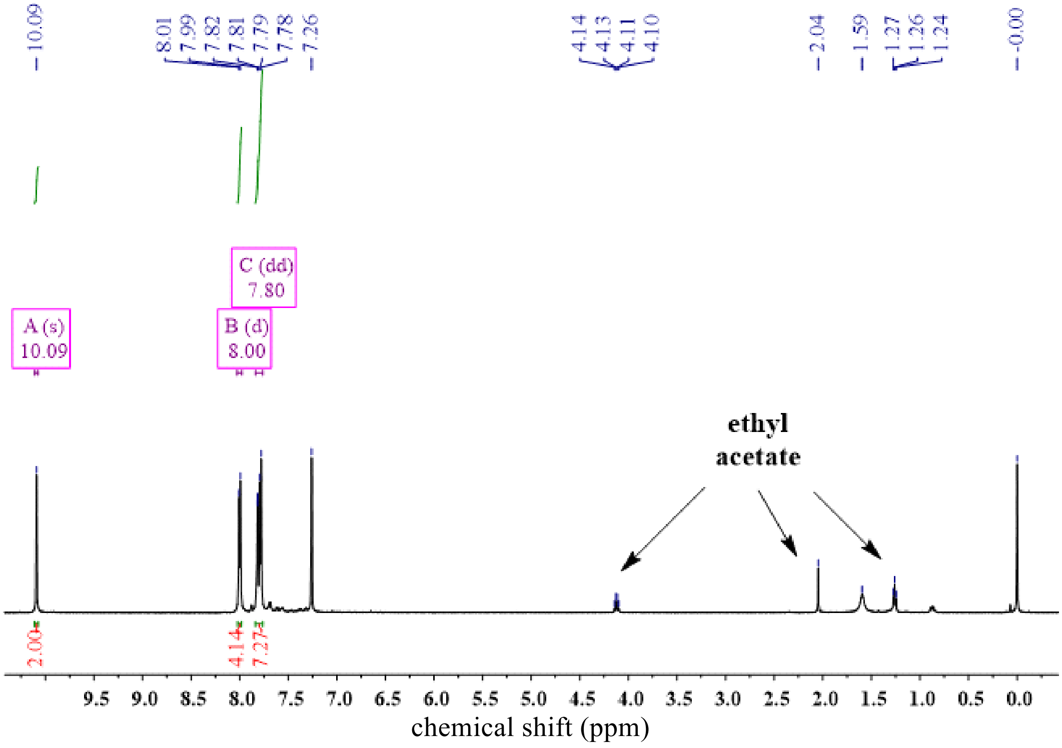

Supplementary Figure 24. ^{1}H NMR (500 MHz, $CDCl_3$) spectrum of 2b.

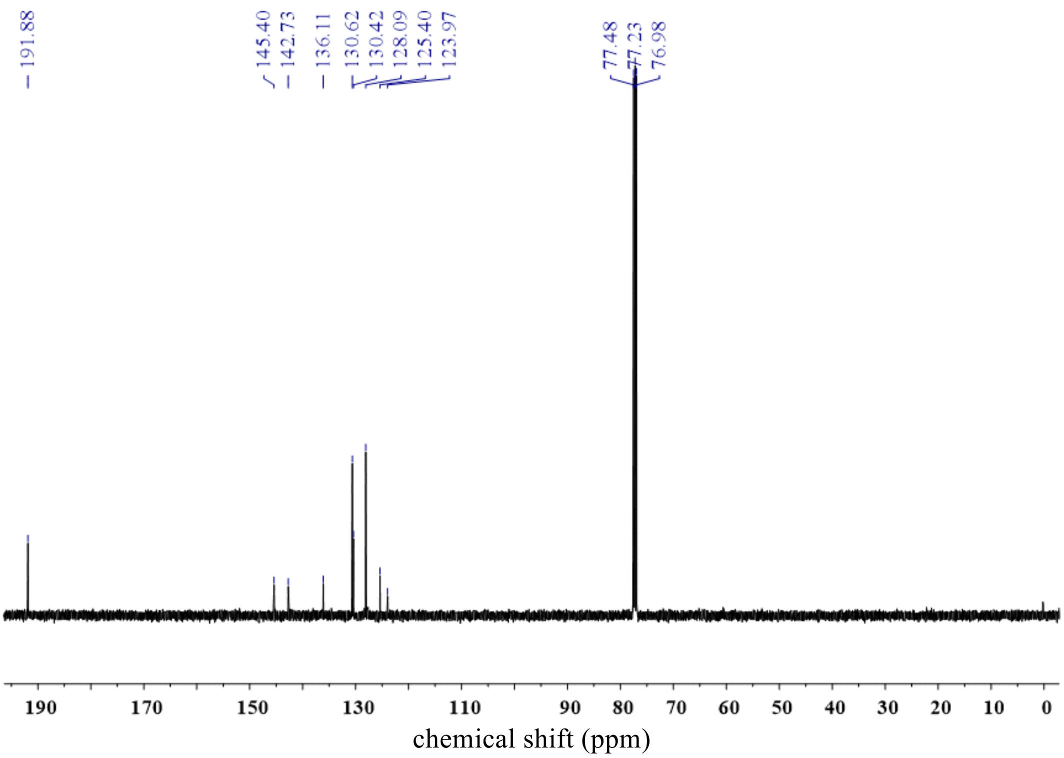

Supplementary Figure 25. ^{13}C NMR (126 MHz, $CDCl_3$) spectrum of 2b.

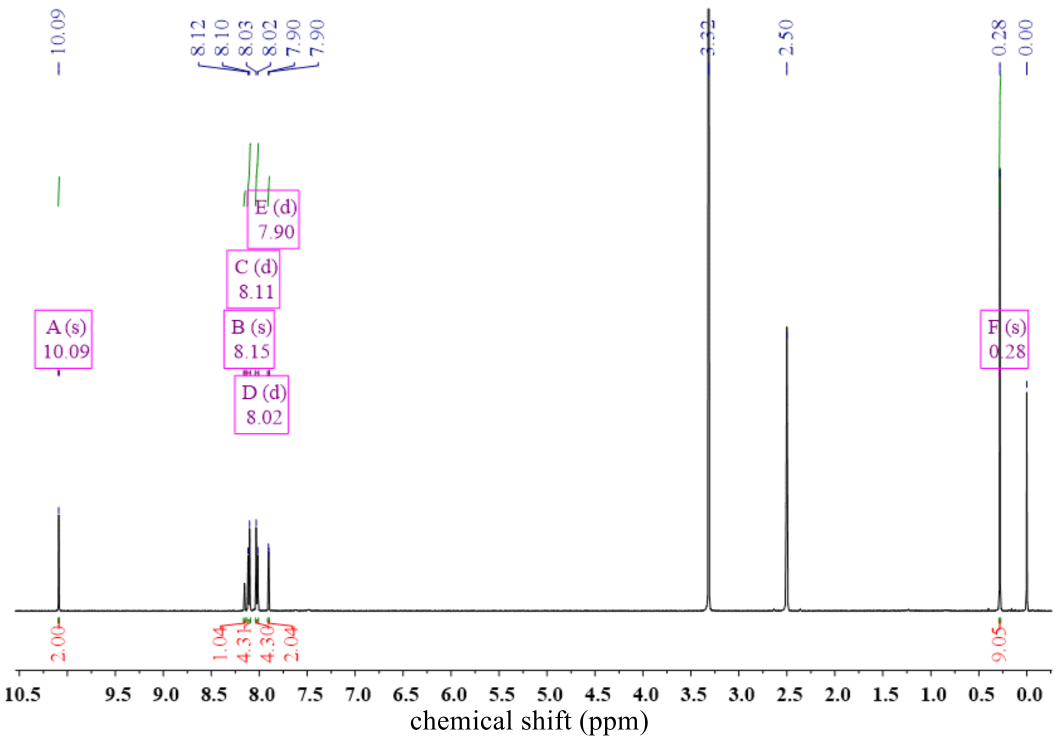

Supplementary Figure 26. ^{1}H NMR (500 MHz, DMSO-d_6) spectrum of 2c.

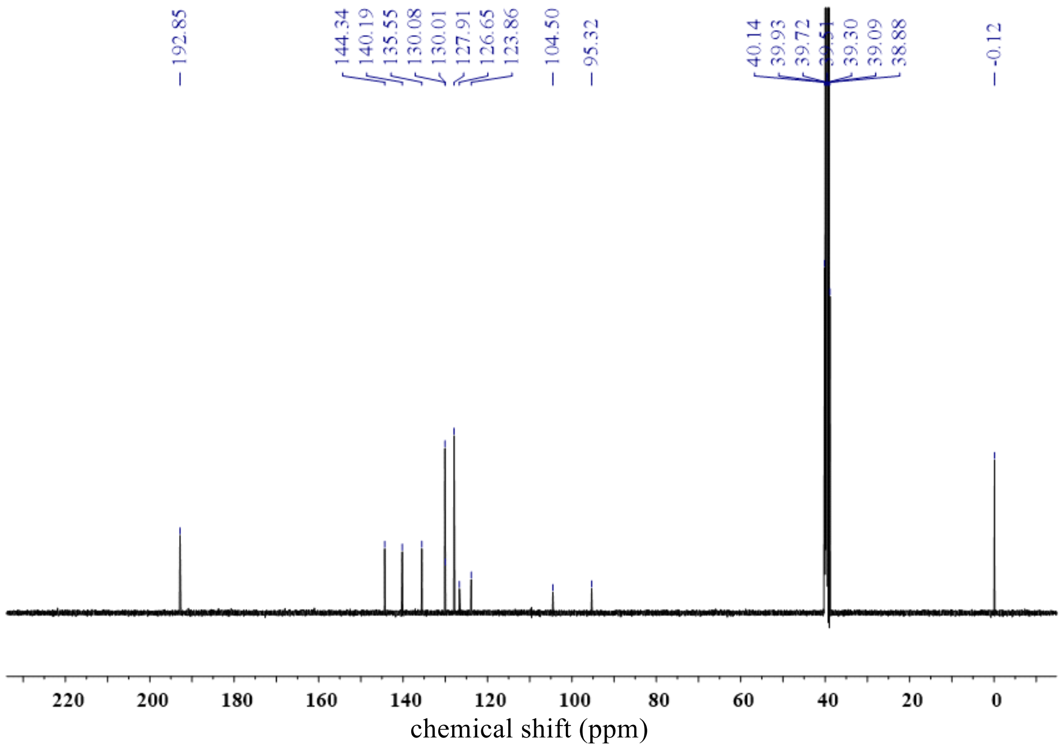

Supplementary Figure 27. ^{13}C NMR (101 MHz, DMSO-d_6) spectrum of 2c.

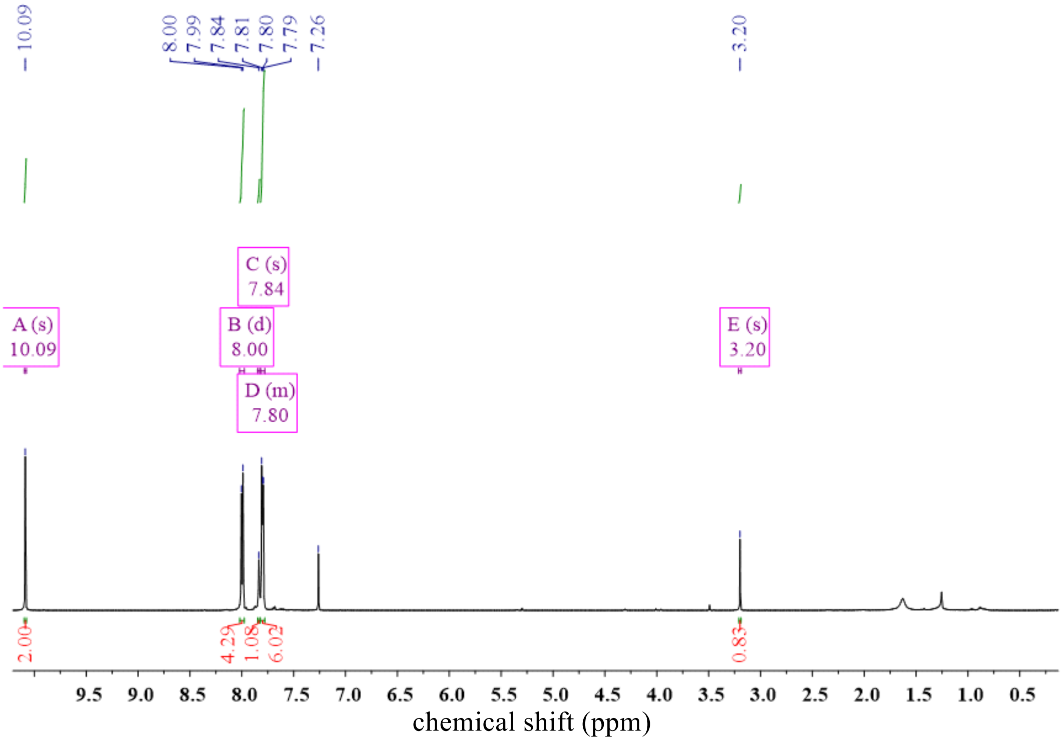

Supplementary Figure 28. ^{1}H NMR (500 MHz, $CDCl_3$) spectrum of 2d.

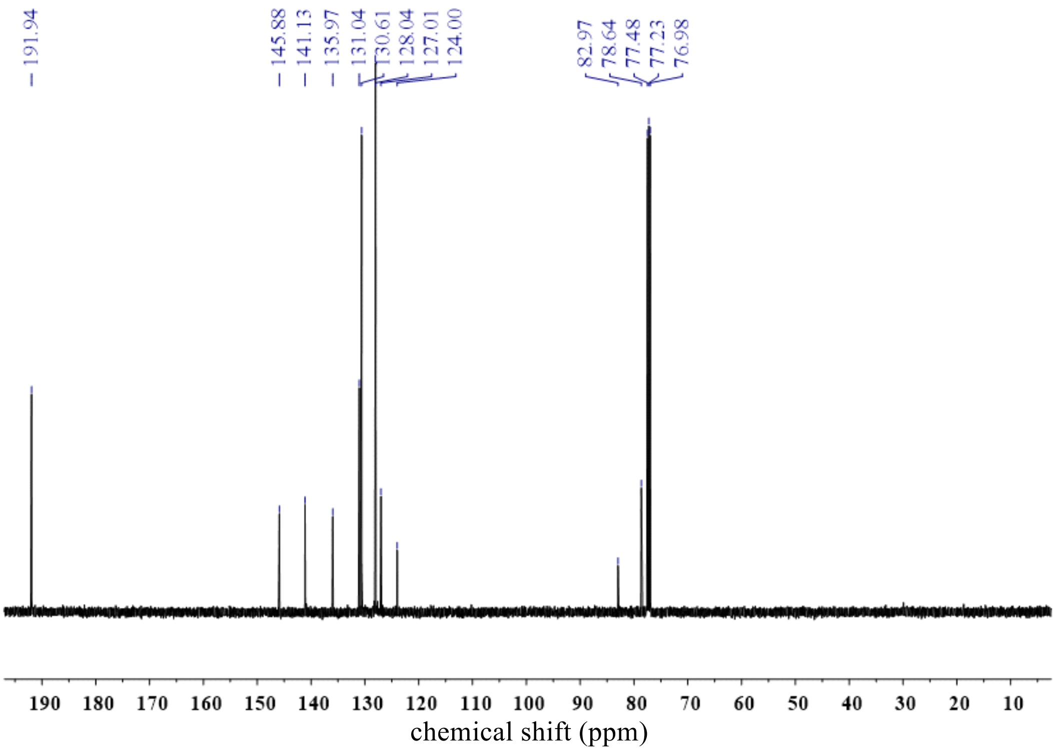

Supplementary Figure 29. ^{13}C NMR (126 MHz, $CDCl_3$) spectrum of 2d.

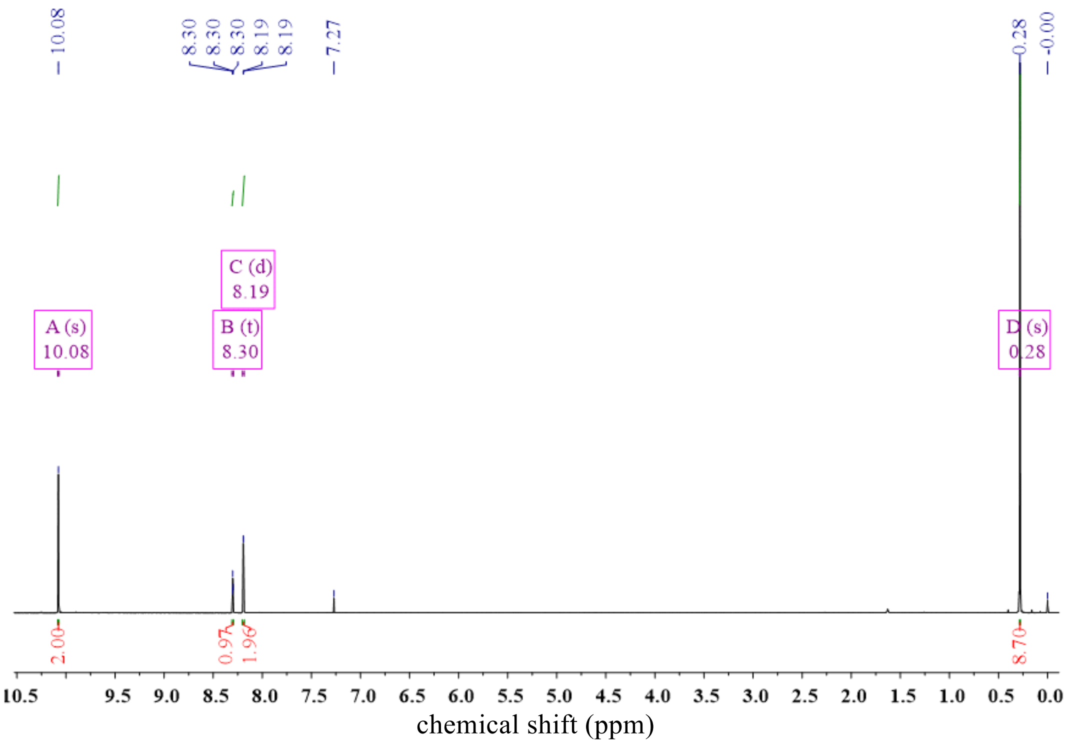

Supplementary Figure 30. ^{1}H NMR (500 MHz, $CDCl_3$) spectrum of 3b.

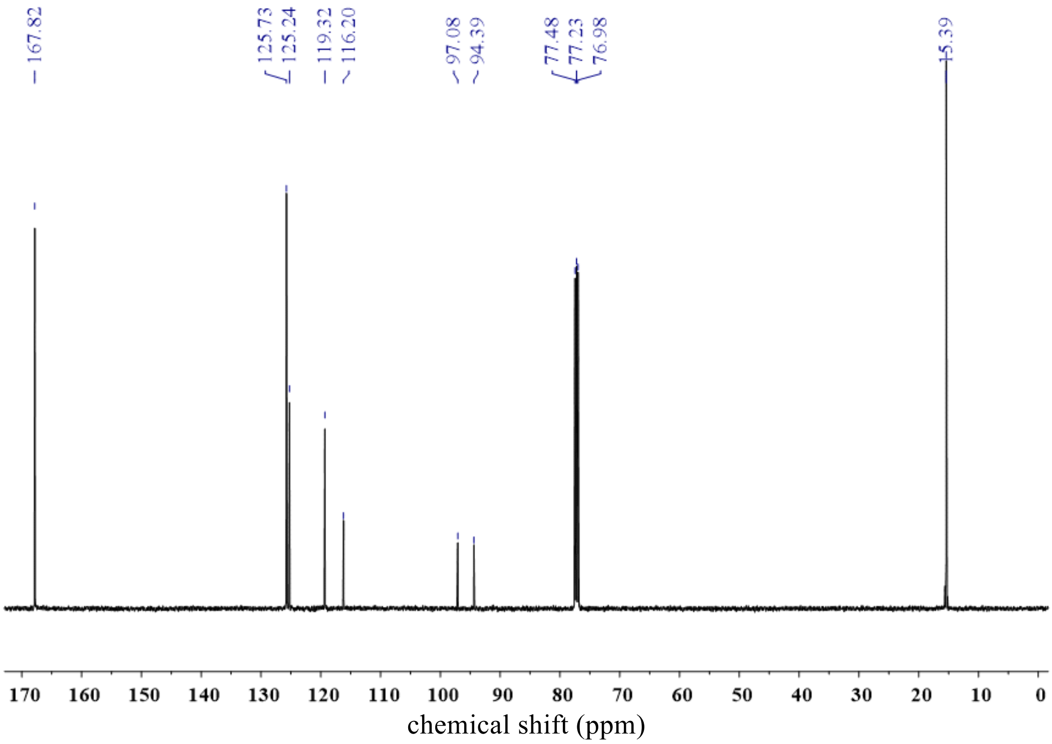

Supplementary Figure 31. ^{13}C NMR (101 MHz, $CDCl_3$) spectrum of 3b.

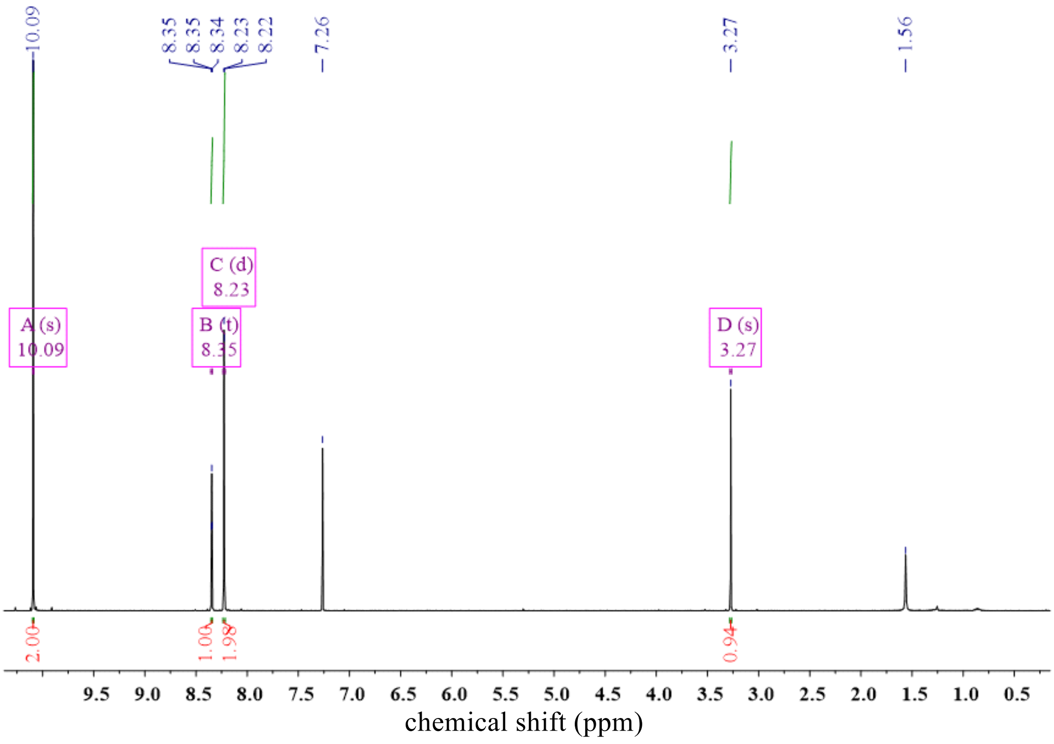

Supplementary Figure 32. ^{1}H NMR (500 MHz, $CDCl_3$) spectrum of 3c.

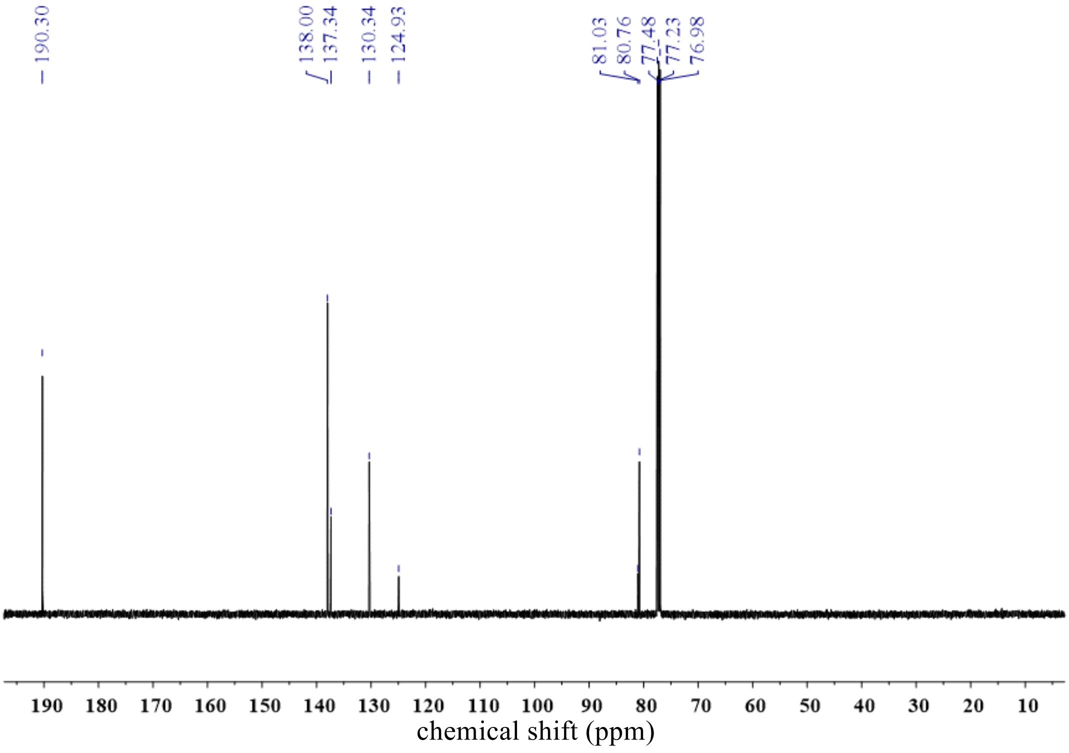

Supplementary Figure 33. ^{13}C NMR (126 MHz, $CDCl_3$) spectrum of 3c.

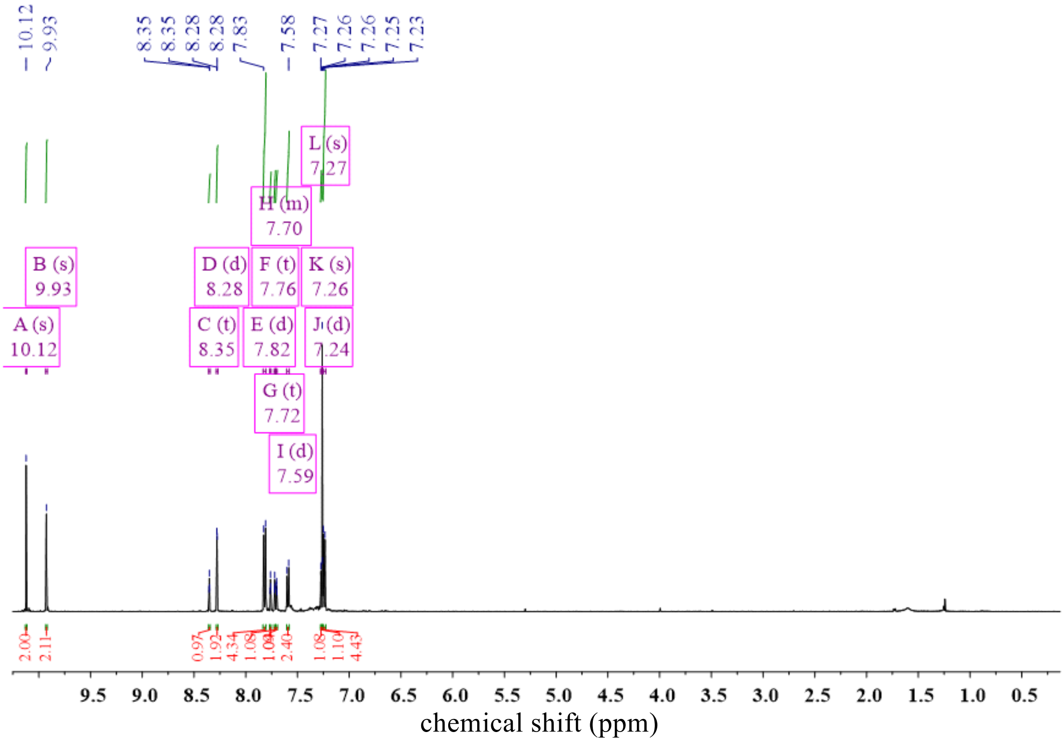

Supplementary Figure 34. ^{1}H NMR (500 MHz, $CDCl_3$) spectrum of 4a.

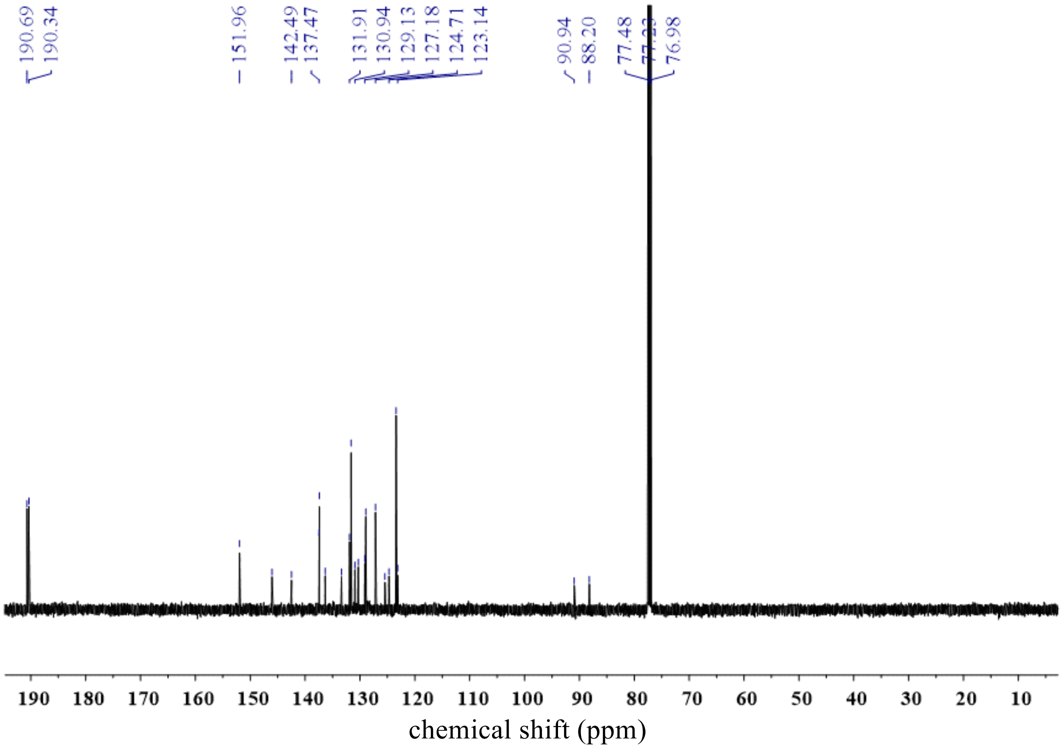

Supplementary Figure 35. ^{13}C NMR (126 MHz, $CDCl_3$) spectrum of 4a.

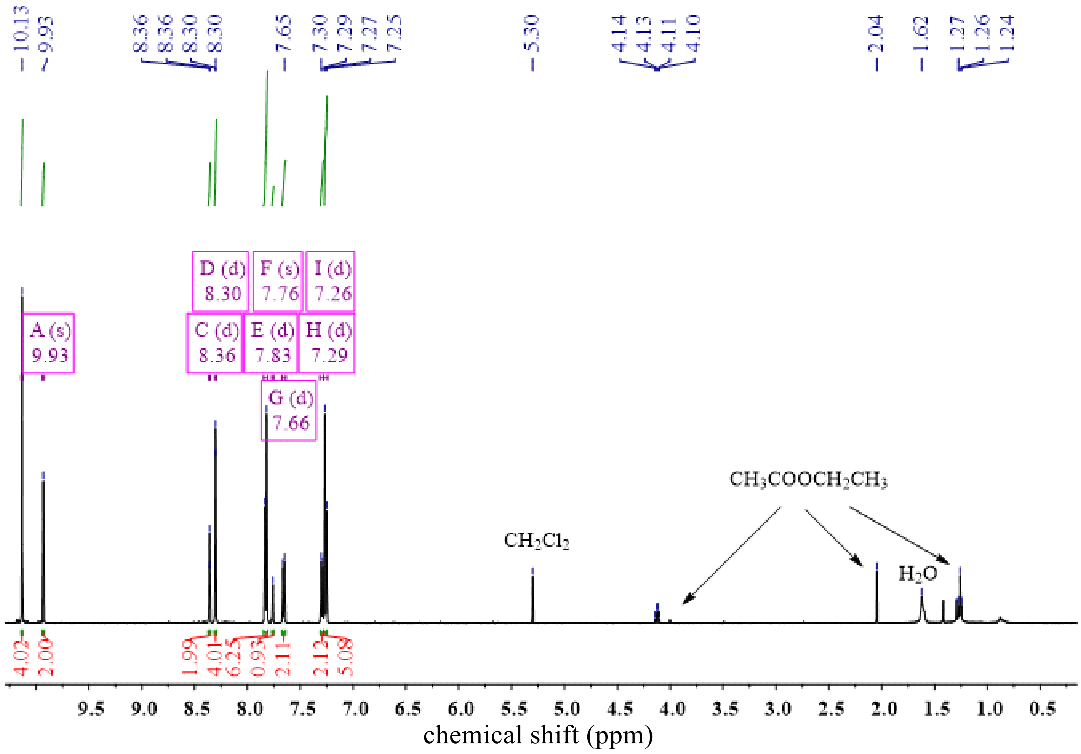

Supplementary Figure 36. ^{1}H NMR (500 MHz, $CDCl_3$) spectrum of TPM.

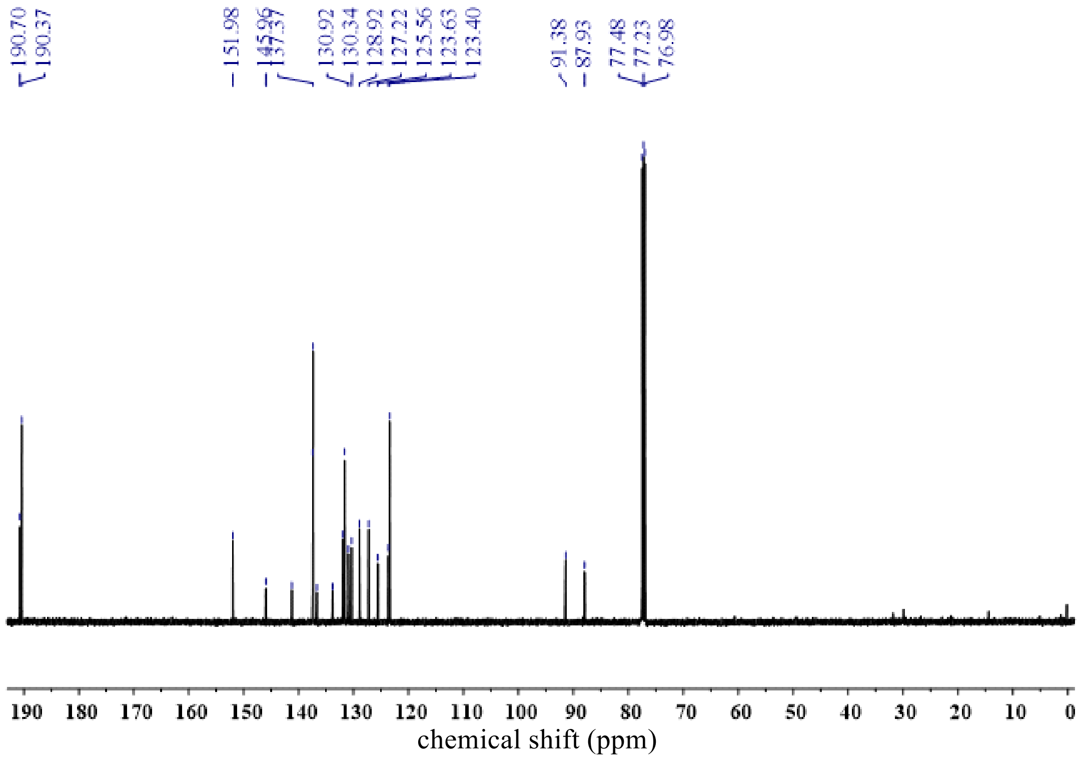

Supplementary Figure 37. ^{13}C NMR (126 MHz, $CDCl_3$) spectrum of TPM.

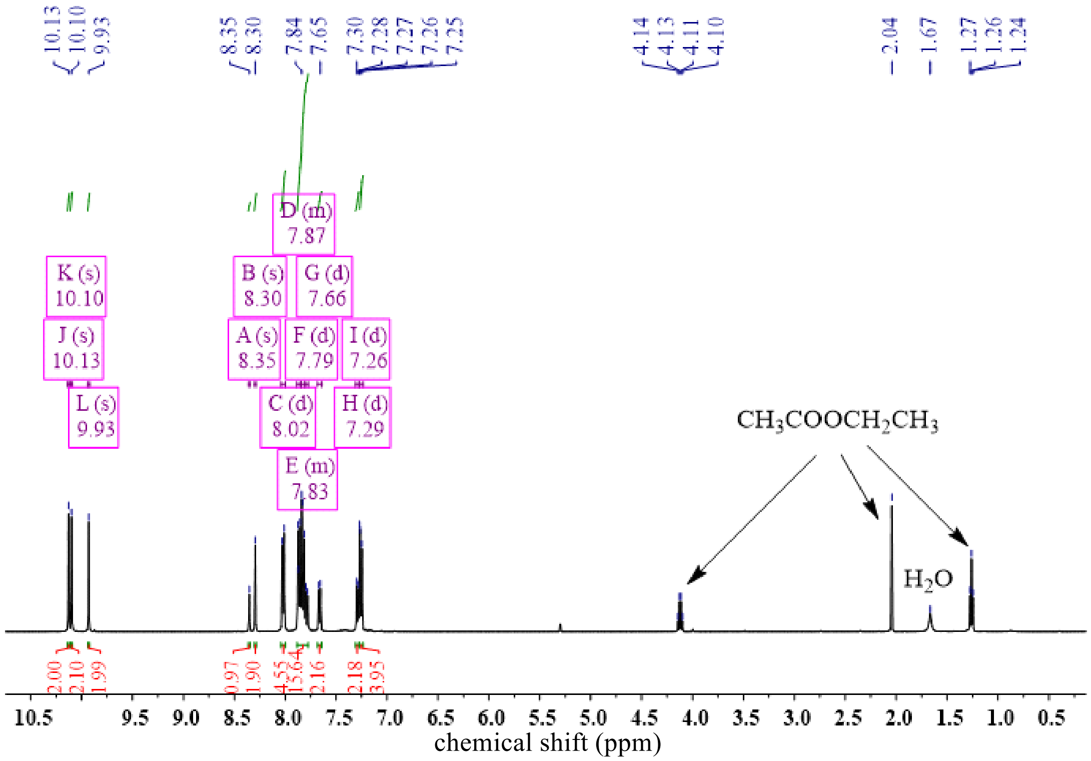

Supplementary Figure 38 ^{1}H NMR (500 MHz, $CDCl_3$) spectrum of FPM.

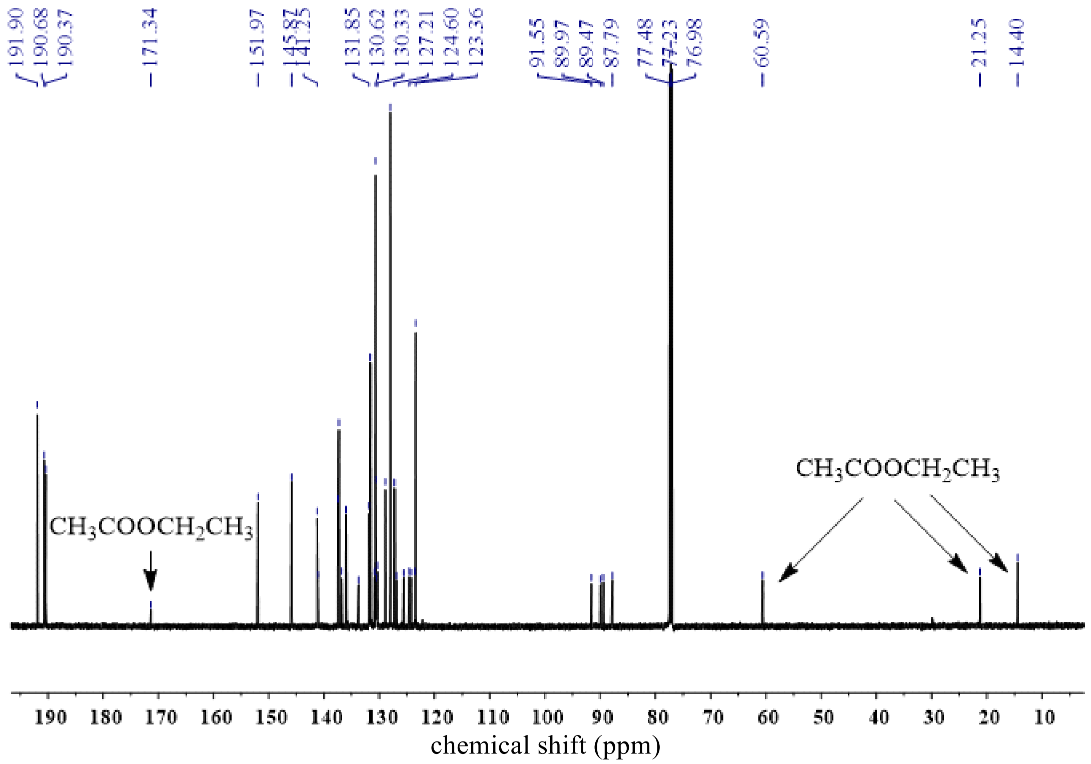

Supplementary Figure 39. ^{13}C NMR (126 MHz, $CDCl_3$) spectrum of FPM.

Supplementary References

1. Zhao, Y. *et al.* A novel donor-acceptor molecule containing a cyclic triphenylamine dimer: synthesis, characterization, and application in memory device. *Tetrahedron* **68**, 1547-1551 (2012).
